# Synthesis and Biological Evaluation of Hydroxylated Monocarbonyl Curcumin Derivatives as Potential Inducers of Neprilysin Activity

**DOI:** 10.3390/biomedicines9080955

**Published:** 2021-08-03

**Authors:** Dimitris Matiadis, See-Ting Ng, Eric H.-L. Chen, Georgia Nigianni, Veroniki P. Vidali, Aleksander Canko, Rita P.-Y. Chen, Marina Sagnou

**Affiliations:** 1 NCSR “Demokritos”, Institute of Biosciences and Applications, Patr. Grigoriou and Neapoleos 27, 153 41 Athens, Greece; matiadis@bio.demokritos.gr (D.M.); gwgwchem@gmail.com (G.N.); 2 Institute of Biological Chemistry, Academia Sinica, No. 128, Sec. 2, Academia Rd., Nankang, Taipei 115, Taiwan; jenny-nst87@hotmail.com (S.-T.N.); hlchan@gate.sinica.edu.tw (E.H.-L.C.); 3 NCSR “Demokritos”, Institute of Nanoscience and Nanotechnology, Patr. Grigoriou and Neapoleos 27, 153 41 Athens, Greece; v.vidali@inn.demokritos.gr (V.P.V.); alexcanko1993@gmail.com (A.C.); 4 Institute of Biochemical Sciences, National Taiwan University, No. 1, Sec. 4, Roosevelt Rd., Taipei 106, Taiwan; 5 Neuroscience Program of Academia Sinica, No. 128, Sec. 2, Academia Rd., Nankang, Taipei 115, Taiwan

**Keywords:** monocarbonyl curcumin derivatives, neprilysin, amyloid cleavage, Aβ amyloid peptide, Alzheimer’s disease

## Abstract

Background: Alzheimer’s disease (AD) involves impairment of Aβ clearance. Neprilysin (NEP) is the most efficient Aβ peptidase. Enhancement of the activity or expression of NEP may provide a prominent therapeutic strategy against AD. Aims: Ten hydroxylated monocarbonyl curcumin derivatives were designed, synthesized and evaluated for their NEP upregulating potential using sensitive fluorescence-based Aβ digestion and inhibition assays. Results: Compound **4** was the most active one, resulting in a 50% increase in Aβ cleavage activity. Cyclohexanone-bearing derivatives exhibited higher activity enhancement compared to their acetone counterparts. Inhibition experiments with the NEP-specific inhibitor thiorphan resulted in dramatic cleavage reduction. **Conclusion:** The increased Aβ cleavage activity and the ease of synthesis of **4** renders it an extremely attractive lead compound.

## 1. Introduction

For almost three decades, there has been an intensely growing and still unmet need to develop clinically successful therapeutics for the treatment of dementia and Alzheimer’s disease (AD) [1]. Since 1992, when it was initially formulated [2], the amyloid cascade hypothesis has been driving the vast majority of therapeutic development in AD [3]. It postulates that the initial pathological event for AD neurodegeneration is the deposition of β-amyloid (Aβ). More specifically, abnormal accumulation of the peptides Aβ40 and Aβ42 can proceed via self-propagation into the aggregation of many different sizes, including soluble intermediate species (oligomers and protofibrils) and insoluble fibrils, which constitute the amyloid plaques. These species are associated with neuronal toxicity and synaptic dysfunction, eventually leading to neuronal death [4]. These peptides are derived either from enhanced amyloidogenic processing of the amyloid precursor protein (APP) or from declined clearance mechanisms. Consequently, Aβ neuronal loading could be reduced by fine-tuning the dynamic equilibrium between the peptide production and clearance. It is noteworthy that the failure of clearance mechanisms rather than the overproduction of the peptide appears to be the predominant cause of peptide accumulation in late-onset (sporadic) forms of the disease [5].

There are two distinct mechanisms primarily responsible for Aβ clearance. The first is the conveyance from the brain to the periphery with subsequent proteolytic removal and the second is the cerebral protease-mediated intra- and/or extracellular hydrolysis [6]. An overwhelming amount of evidence suggests the age- and pathology-related decrease in the expression or the activity of Aβ-degrading enzymes, such as the insulin-degrading enzyme (IDE) and neprilysin (NEP) [7]. More specifically, prodromal and early AD stages have been associated with low NEP levels, whereas an increased amount of Aβ was observed upon alterations in NEP expression [8,9,10]. Furthermore, synaptic plasticity and cognitive functions were significantly compromised in hAPP mouse models as a result of NEP inactivation [11]. Additionally, colocalisation of amyloid plaques with increased NEP levels was observed in transgenic Tg2576 Alzheimer-like mouse brain [12]. Finally, in a very recent report, the intravenous injections of a blood–brain barrier-permeable somatostatin-like peptide resulted in a significant increase in NEP levels, which was also accompanied by a significant and selective degradation of membrane-bound Aβ42 in the hippocampus [4]. Consequently, the upregulation of NEP can be considered a promising strategy against AD [13,14].

**Figure 1:**
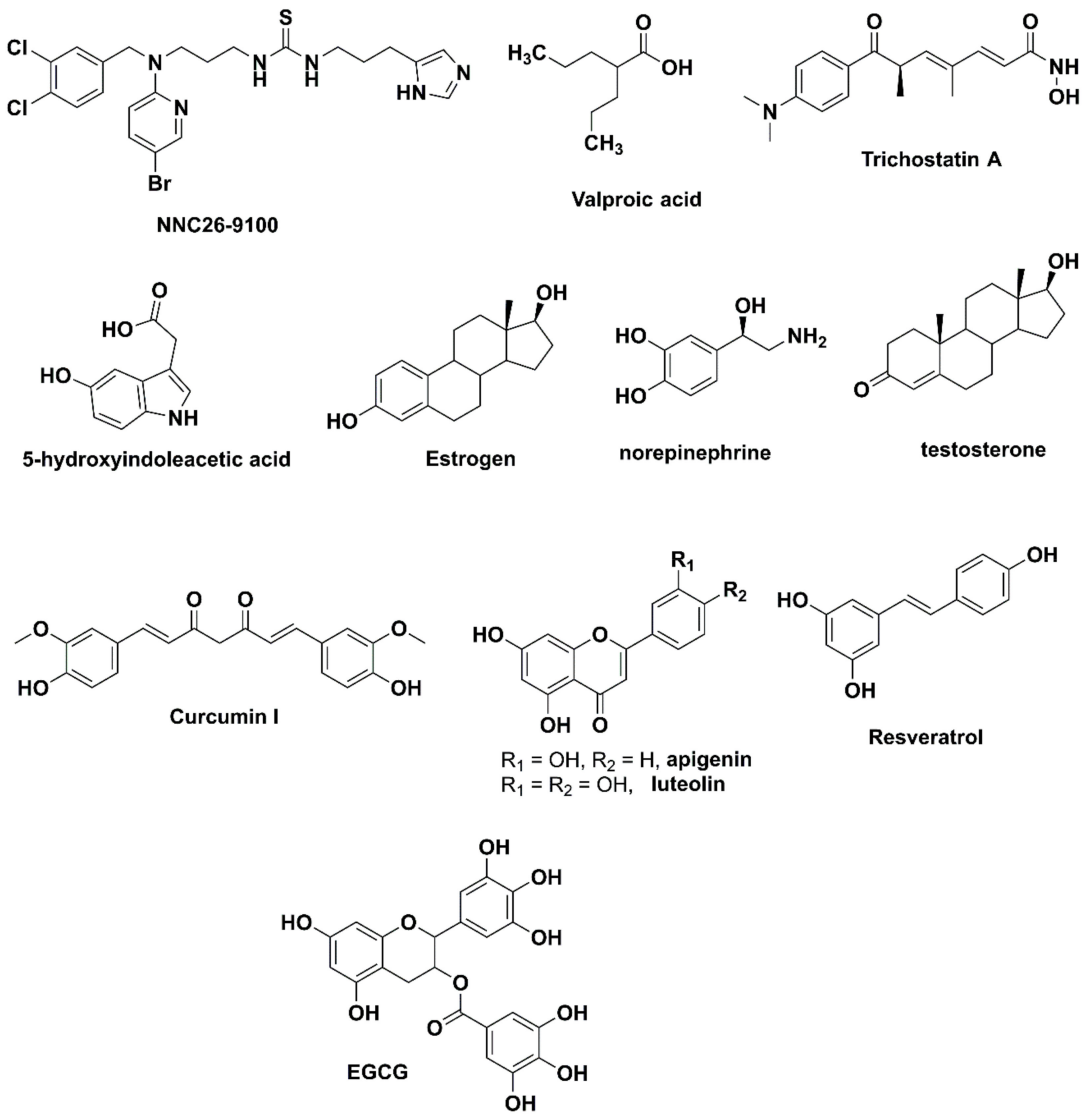
The structural variation of molecules studied as NEP enhancers.

To this end, a variety of natural and synthetic molecules have been proposed as potential agonists aiming to increase either the expression levels or the hydrolytic function in in vitro or in vivo research protocols. For example, the thiourea NNC26-9100 (Figure 1) decreased APP expression, resulting in the reduction in Aβ formation, and it inhibited the formation of Aβ42 trimers within both extracellular and intracellular cortical fractions [15,16]. The widely used histone deacetylase inhibitors, valproic acid and trichostatin A, exhibited significant ability to enhance the expression of NEP in SHSY-5Y cells, as well as in the cortex and hippocampus of rats [17,18]. Furthermore, 5-hydroxyindoleacetic acid (5-HIAA) caused upregulation of the levels of NEP in neuroblastoma cells and in vivo [19]. Estrogen, norepinephrine and testosterone induced elevation of NEP expression, leading to the amelioration of cognitive deficits in AD animal models [20]. With regard to natural products, EGCG strongly increased the NEP activity, thus leading to Aβ degradation [21], and resveratrol significantly increased both the estradiol and NEP level, thus decreasing Aβ deposition [22]. Finally, apigenin, luteolin and curcumin were able to induce specific NEP activity in SK-N-SH cells [23,24]. Recently, the work of Rita P-Y Chen and her group identified four curcuminoid compounds, the dihydroxylated curcumin, the monohydroxylated demethoxycurcumin, and the mono- and di-hydroxylated bisdemethoxycurcumin that have the ability to increase NEP activity [25]. Moreover, it has been found that curcumin has very poor solubility and bioavailability, whereas demethoxylation improves solubility and stability [26].

Herein, we would like to report the design, synthesis and biological evaluation of some hydroxylated monocarbonyl curcumin derivatives for the prevention and therapy of Alzheimer’s disease through the upregulation of NEP-related activity. The potency of the compounds to induce NEP activity was evaluated by means of sensitive fluorescence-based Aβ digestion assays and inhibition assays.

## 2. Materials and Methods

### 2.1. General

All reagents were purchased from Sigma-Aldrich (St. Louis, MO, USA), Alfa Aesar (Lancaster, UK) and TCI (Tokyo, Japan) and used without further purification. NMR spectra were recorded with a Bruker Avance 500 MHz spectrometer (Bruker, Rheinstetten, Germany) operating at 500 MHz (^1^H) and 125 MHz (^13^C) or with a Bruker Avance 250 MHz spectrometer (Bruker, Rheinstetten, Germany) operating at 250 MHz (^1^H) and 62.5 MHz (^13^C). Chemical shifts are reported in ppm relative to DMSO-*d*_6_ (^1^H: δ = 2.50 ppm, ^13^C: δ = 39.52 ± 0.06 ppm). Elemental analyses were performed using a PerkinElmer 2400 CHNS 100V Organic Elemental Analyzer (Waltham, MA, USA). Compounds **5–7** were synthesised as described previously [27,28,29].

### 2.2. Synthesis of Compounds

#### 2.2.1. General Procedure for Preparation of Compounds **1**–**4**

Acetone (0.73 mL, 10 mmol) and concentrated hydrochloric acid (0.2 mL) were added to a suspension of 4-hydroxybenzaldehyde (2.44 g, 20 mmol) or vanillin (3.04 g, 20 mmol) in ethanol (3 mL). The reaction mixture was stirred at room temperature for 24 h. Upon completion of the reaction, the reaction mixture was concentrated under vacuum to half volume, poured into ice-cold water (40 mL), and 1% aqueous KOH solution was added until pH 6–7. Then, the precipitate was filtered and washed with water (×2) and hot water (×2) for **1** and **4** or only with water (<35 °C, ×2) for **2** and **3**. Compound **3** was recrystallized from ethanol.

(1*E*,4*E*)-1,5-bis(4-hydroxyphenyl)penta-1,4-dien-3-one **1** [30]

Yield: 1.87 g (70%), ^1^H NMR (500 MHz, DMSO-*d*_6_): 6.83 (4H, d, J = 8.4 Hz, Ar-*H*), 7.10 (2H, d, J = 16.0 Hz, C*H*C=O), 7.62 (4H, d, J = 8.4 Hz, Ar-*H*), 7.66 (2H, J = 16.0 Hz, C*H*Ar), 10.03 (2H, s, OH). Anal. Calc. for C_17_H_14_O_3_: C 76.86, H 5.30. Found: C 76.58, H 5.24.

(1*E*,4*E*)-1,5-bis(4-hydroxy-3-methoxyphenyl)penta-1,4-dien-3-one **2** [30]

Yield: 2.15 g (66%), ^1^H NMR (500 MHz, DMSO-*d*_6_): 3.85 (6H, s, C*H_3_*O), 6.83 (2H, d, J = 8.2 Hz, Ar-*H*), 7.15 (2H, d, J = 16.0 Hz, C*H*C=O), 7.20 (2H, d, J = 8.2 Hz, Ar-*H*), 7.37 (2H, s, Ar-*H*), 7.65 (2H, d, J = 16.0 Hz, C*H*Ar), 9.66 (2H, s, OH); ^13^C NMR (125 MHz, DMSO-*d*_6_): 55.7 (*C*H_3_O), 111.4 (Ar), 115.6 (Ar), 123.0 (*C*HC=O), 123.3 (Ar), 126.3 (Ar), 142.7 (*C*HAr), 147.9 (Ar), 149.4 (Ar), 188.0 (C=O). Anal. Calc. for C_19_H_18_O_5_: C 69.93, H 5.56. Found: C 69.82, H 5.50.

2,6-bis((*E*)-4-hydroxybenzylidene)cyclohexan-1-one **3 [30]**

Yield: 2.23 g (73%), ^1^H NMR (500 MHz, DMSO-*d*_6_): 1.67–1.75 (2H, m, CH_2_C*H_2_*CH_2_), 2.82–2.88 (4H, m, C*H_2_*CH_2_C*H_2_*), 6.84 (4H, d, J = 8.4 Hz, Ar-*H*), 7.41 (4H, d, J = 8.4 Hz, Ar-*H*), 7.54 (2H, s, C*H*Ar), 9.93 (2H, s, OH). Anal. Calc. for C_20_H_18_O_3_: C 78.41, H 5.92. Found: C 78.33, H 5.83.

2,6-bis((*E*)-4-hydroxy-3-methoxybenzylidene)cyclohexan-1-one **4 [30]**

Yield: 2.55 g (70%), ^1^H NMR (500 MHz, DMSO-*d*_6_): 1.70–1.75 (2H, m, CH_2_C*H_2_*CH_2_), 2.88–2.91 (4H, m, C*H_2_*CH_2_C*H_2_*) 3.81 (6H, s, C*H_3_*O), 6.85 (2H, d, J = 8.2 Hz, Ar-*H*), 7.03 (2H, d, J = 8.2 Hz, Ar-*H*), 7.11 (2H, s, Ar-*H*), 7.56 (2H, s, C*H*Ar), 9.53 (2H, s, OH). Anal. Calc. for C_22_H_22_O_5_: C 72.12, H 6.05. Found: C 72.04, H 6.08.

#### 2.2.2. General Procedure for Preparation of Compounds **8–10**

The appropriate intermediate **5**–**7** (0.5 mmol) and anhydrous DCM (10 mL) were added in a 50 mL two-necked round bottom flask under N_2_. The solution was cooled to -20 °C using ice/NaCl bath. Boron tribromide (2.5 mmol for **8** and **9**, 3.5 mmol for **10**) was injected carefully with a syringe. The reaction mixture was stirred for 1 h at −20 °C, then for 1 h at 0 °C and for 1 h at room temperature. Upon completion of the reaction, ice-cold water was poured into the mixture, and the flask was shaken for a few minutes. The resulting precipitate was filtered and washed with small amounts of water. An additional amount of product was recovered from the filtrate after extraction with EtOAc.

(1*E*,4*E*)-1,5-bis(3,4-dihydroxyphenyl)penta-1,4-dien-3-one **8** [31]

Yield: 79 mg (53%), ^1^H NMR (500 MHz, DMSO-*d*_6_): 6.79 (2H, d, *J* = 8.2 Hz, Ar-*H*), 6.99 (2H, d, *J* = 16.0 Hz, C*H*C=O), 7.07 (2H, d, *J* = 8.2 Hz, Ar-*H*), 7.14 (2H, s, Ar-*H*), 7.56 (2H, *J* = 16.0 Hz, C*H*Ar), 9.38 (4H, br, OH), Anal. Calc. for C_17_H_14_O_5_: C 68.45, H 4.73. Found: C 68.32, H 4.66.

(2*E*,6*E*)-2,6-bis(3,4-dihydroxybenzylidene)cyclohexanone **9** [31]

Yield: 120 mg (71%), ^1^H NMR (500 MHz, DMSO-*d*_6_): 1.69–1.74 (2H, m, CH_2_C*H_2_*CH_2_), 2.83–2.86 (4H, m, C*H_2_*CH_2_C*H_2_*), 6.80 (2H, d, *J* = 8.2 Hz, Ar-*H*), 6.87 (2H, d, *J* = 8.2 Hz, Ar-*H*), 6.98 (2H, s, Ar-*H*), 7.45 (2H, s, C*H*Ar), 9.13 (1H, s, OH), 9.44 (1H, s, OH), Anal. Calc. for C_20_H_18_O_5_: C 70.99, H 5.36. Found: C 71.04, H 5.28.

(1*E*,4*E*)-1,5-bis(3,4,5-trihydroxyphenyl)penta-1,4-dien-3-one **10**

Yield: 74 mg (45%), ^1^H NMR (500 MHz, DMSO-*d*_6_): 6.69 (4H, s, Ar-*H*), 6.91 (2H, d, J = 8.4 Hz, C*H*C=O), 7.45 (2H, d, J = 8.4 Hz, C*H*Ar), 8.95 (2H, s, OH), 9.09 (4H, s, OH); ^13^C NMR (62.5 MHz, DMSO-*d*_6_): 108.0 (Ar), 122.8 (*C*HC=O), 125.2 (Ar), 136.5 (Ar), 143.2 (*C*HAr), 146.2 (Ar), 187.8 (C=O), Anal. Calc. for C_17_H_14_O_7_: C 61.82, H 4.27. Found: C 61.74, H 4.31.

#### 2.2.3. General Procedure for Preparation of Compounds **11**, **15** and **18**

An aqueous solution of NaOH 1M (1 mL) was added to a solution of veratraldehyde (1.66 g, 10 mmol) or vanillin (1.52 g, 10 mmol) or 3,4,5-trimethoxybenzaldehyde (1.96 g, 10 mmol) in acetone (30 mL). The reaction mixture was stirred for 24 h at room temperature. Upon completion of the reaction, the solution was concentrated under vacuum to 1/3 of its volume. The specific work-up procedures are described below.

(*E*)-4-(3,4-dimethoxyphenyl)but-3-en-2-one **11** [32]

Yellow solid was precipitated after addition of ice-cold water (30 mL) and overnight cooling at 4 °C. The precipitate was filtered, washed with water and dried under vacuum

Yield: 1.14 g (55%), ^1^H NMR (500 MHz, DMSO-*d*_6_): 2.30 (3H, s, C*H_3_*C=O), 3.80 (3H, s, C*H_3_*O), 3.81 (3H, s, C*H_3_*O), 6.74 (1H, d, *J* = 16.2 Hz, C*H*Ar), 7.01 (1H, d, *J* = 8.4 Hz, Ar-*H*), 7.26 (1H, d, *J* = 8.4 Hz, Ar-*H*), 7.32 (1H, s, Ar-*H*), 7.56 (1H, d, *J* = 16.2 Hz, C*H*C=O).

(*E*)-4-(4-hydroxy-3-methoxyphenyl)but-3-en-2-one **15** [32]

Yellow solid was precipitated after acidification with HCl 10%. The precipitate was filtered, washed with water and dried under vacuum.

Yield: 1.63 g (85%), ^1^H NMR (500 MHz, DMSO-*d*_6_): 2.29 (3H, s, C*H_3_*C=O), 3.82 (3H, s, C*H_3_*O), 6.67 (1H, d, *J* = 16.2 Hz, C*H*Ar), 6.81 (1H, d, *J* = 8.2 Hz, Ar-*H*), 7.13 (1H, d, *J* = 8.2 Hz, Ar-*H*), 7.30 (1H, s, Ar-*H*), 7.52 (1H, d, *J* = 16.2 Hz, C*H*C=O), 9.63 (1H, s, OH).

(*E*)-4-(3,4,5-trimethoxyphenyl)but-3-en-2-one **18 [33]**

Brown oil was formed after addition of ice-cold water. The product was isolated by flash chromatography (CHCl_3_) as an off-white solid.

Yield: 1.04 g (44%), ^1^H NMR (500 MHz, DMSO-*d*_6_): 2.32 (3H, s, C*H_3_*C=O), 3.70 (3H, s, C*H_3_*O), 3.82 (6H, s, C*H_3_*O), 6.82 (1H, d, *J* = 16.4 Hz, CHC=O), 7.06 (2H, s, Ar-*H*), 7.56 (1H, d, *J* = 16.4 Hz, C*H*Ar).

#### 2.2.4. Preparation of Compound **12**

*p*-Anisaldehyde (1.33 g, 9.7 mmol) and ketone **11** in EtOH (2 mL) were added dropwise to a solution of NaOH (0.78 g, 19.4 mmol) in EtOH (14 mL) and water (14 mL). After stirring for 16 h at room temperature, a beige oil was formed. After separation of the oily product from the upper layer, HCl 1M and EtOAc were added, and the two phases were separated. The aqueous layer was extracted two more times with EtOAc. The organic extract was washed with HCl 1M (×2) and brine, and then dried with NaSO_4_ and concentrated in vacuo to give an orange coloured oil. The yellow coloured pure product was isolated by flash chromatography (DCM to DCM:EtOAc = 8/2).

(1*E*,4*E*)-1-(3,4-dimethoxyphenyl)-5-(4-methoxyphenyl)penta-1,4-dien-3-one **12** [34]

Yield: 1.10 g (35%), ^1^H NMR (500 MHz, DMSO-*d*_6_): 3.81 (3H, s, C*H_3_*O), 3.82 (3H, s, C*H_3_*O), 3.84 (3H, s, C*H_3_*O), 7.02 (2H, d, *J* = 8.6 Hz, Ar-*H*), 7.03 (1H, d, *J* = 8.2 Hz, Ar-*H*), 7.21 (1H, d, *J* = 16.0 Hz, C*H*C=O), 7.22 (1H, d, *J* = 16.0 Hz, C*H*C=O), 7.33 (1H, d, *J* = 8.6 Hz, Ar-*H*), 7.40 (1H, s, Ar-*H*), 7.69–7.75 (4H, m, C*H*Ar, Ar-*H*). ^13^C NMR (125 MHz, DMSO-*d*_6_): 55.3 (*C*H_3_O), 55.57 (*C*H_3_O), 55.60 (*C*H_3_O), 110.6 (Ar), 111.6 (Ar), 114.4 (Ar), 123.1 (*C*HC=O), 123.4 (*C*HC=O), 123.9 (Ar), 127.4 (Ar), 127.6 (Ar), 130.2 (Ar), 142.0 (*C*HAr), 142.6 (*C*HAr), 149.0 (Ar), 151.0 (Ar), 161.1 (Ar), 188.1 (C=O).

#### 2.2.5. Preparation of Compound **13**

Intermediate compound **12** (162 mg, 0.5 mmol) and anhydrous DCM (10 mL) were added in a 50 mL two-necked round bottom flask under N_2_. The solution was cooled to -20 °C using ice/NaCl bath. Boron tribromide (2.0 mmol) was injected carefully with a syringe. The reaction mixture was stirred for 1 h at −20 °C, then for 1 h at 0 °C and 1 h at room temperature. Upon completion of the reaction, ice-cold water was poured into the mixture, and the flask was shaken for a few minutes. The mixture was extracted with Et_2_O (×3). Then, the combined organic extracts were extracted with a 10% aqueous solution of NaOH. The aqueous solution was acidified with an aqueous solution of HCl 10% until pH 5 and then extracted again with Et_2_O (×3). The organic extracts were dried with Na_2_SO_4_ and concentrated in vacuo to give an oily crude product. The final product was isolated by flash chromatography (DCM:MeOH = 98/2 to 90/10) as a dark green powder.

(1*E*,4*E*)-1-(3,4-dihydroxyphenyl)-5-(4-hydroxyphenyl)penta-1,4-dien-3-one **13** [35]

Yield: 48 mg (34%), ^1^H NMR (500 MHz, DMSO-*d*_6_): 6.79 (1H, d, *J* = 8.2 Hz, Ar-*H*), 6.83 (2H, d, *J* = 8.2 Hz, Ar-*H*), 6.97 (1H, d, *J* = 16.0 Hz, C*H*C=O), 7.07 (1H, d, *J* = 8.2 Hz, Ar-*H*), 7.11 (1H, d, *J* = 16.0, C*H*C=O), 7.14 (1H, s, Ar-*H*), 7.58 (1H, d, *J* = 16.0 Hz, C*H*Ar), 7.61–7.64 (3H, 2 × d overlapping, *J* = 8.2 Hz, 16.0 Hz, Ar-*H*, C*H*Ar), 9.60 (3H, br, OH). Anal. Calc. for C_17_H_14_O_4_: C 72.33, H 5.00. Found: C 72.24, H 5.06.

#### 2.2.6. Preparation of Compound **14**

Dry K_2_CO_3_ (11 g, 75 mmol), 3,4-dihydroxybenzaldehyde (2 g, 15 mmol) and dry DMF (15 mL) were added in a round bottom flask under argon. Methoxymethylchloride (4.5 mL, 60 mmol) was added dropwise over 2 h. The reaction mixture was then stirred vigorously for 16 h at room temperature. Upon completion of the reaction, the solvent was evaporated to dryness. After addition of water, the mixture was extracted with CHCl_3_ (×3). The combined extracts were washed with HCl 1M (×2), brine, dried with Na_2_SO_4_ and concentrated to give a beige-coloured oil. The pure product was isolated by flash chromatography (n-hexane:EtOAc = 8/2) as a white solid.

3,4-bis(methoxymethoxy)benzaldehyde **14** [35]

Yield: 2.82 g (83%), ^1^H NMR (500 MHz, DMSO-*d*_6_): 3.40 (3H, s, C*H_3_*OCH_2_O), 3.41 (3H, s, C*H_3_*OCH_2_O), 5.27 (2H, s, CH_3_OC*H_2_*O), 5.33 (2H*,* s, CH_3_OC*H_2_*O), 7.30 (1H, d, *J* = 8.6 Hz, Ar-*H*), 7.57–7.61 (2H, m, Ar-*H*), 9.84 (s, 1H, C*H*O).

#### 2.2.7. Preparation of Compound **17**

Compound **16** was prepared by reacting aldehyde **14** with ketone **15** similarly as described above for **12** (product was isolated by flash chromatography, n-hexane:EtOAc = 6/4). Compound **16** (160 mg, 0.4 mmol) was dissolved in MeOH (5 mL). After dropwise addition of 3 M HCl, the mixture was stirred at 65 °C for 2 h. Upon completion of the reaction, the methanol was evaporated. After extraction of the mixture with EtOAc (×3), the combined organic extracts were washed with brine, dried with Na_2_SO_4_ and concentrated to give a brown-coloured oil. The pure product was isolated by column chromatography (CHCl_3_:MeOH = 9/1) as a bright yellow solid.

(1*E*,4*E*)-1-(3,4-dihydroxyphenyl)-5-(4-hydroxy-3-methoxyphenyl)penta-1,4-dien-3-one **17** [36]

Yield: 56 mg (45%), ^1^H NMR (500 MHz, DMSO-*d*_6_): 3.85 (3H, s, C*H_3_*O), 6.80 (1H, d, *J* = 8.2 Hz, Ar-*H*), 6.83 (1H, d, *J* = 8.2 Hz, Ar-*H*), 7.00 (1H, d, *J* = 15.8 Hz, C*H*C=O), 7.08 (1H, d, *J* = 8.2 Hz, Ar-*H*), 7.13–7.16 (2H, 1 d and 1 s overlapping, J = 15.8 Hz, C*H*C=O, Ar-*H*), 7.20 (1H, d, *J* = 8.2 Hz, Ar-*H*), 7.37 (1H, s, Ar-*H*), 7.58 (1H, *J* = 15.8 Hz, C*H*Ar), 7.64 (1H, *J* = 15.8 Hz, C*H*Ar), 9.59 (4H, br, OH). ^13^C NMR (125 MHz, DMSO-*d*_6_): ^13^C NMR (125 MHz, DMSO-*d*_6_): 55.7 (*C*H_3_O), 111.5 (Ar), 114.9 (Ar), 115.6 (Ar), 115.8 (Ar), 121.6 (Ar), 122.6 (Ar), 123.0 (*C*HC=O), 123.3 (*C*HC=O), 126.31 (Ar), 126.35 (Ar), 142.6 (*C*HAr), 142.8 (*C*HAr), 145.6 (Ar), 147.9 (Ar), 148.4 (Ar), 149.3 (Ar), 187.9 (C=O). Anal. Calc. for C_18_H_16_O_5_: C 69.22, H 5.16. Found: C 69.19, H 5.08.

#### 2.2.8. Synthesis of Compound **19**

Compound **19** was prepared similarly as described above for **8**–**10**. A quantity of 2.0 mmoles of BBr_3_ was used and, during the work-up, after shaking the reaction mixture with water, it was extracted with EtOAc (×3). The organic extracts were dried with Na_2_SO_4_ and the final product was isolated as yellow solid by flash chromatography (DCM:MeOH = 95/5).

(*E*)-4-(3,4,5-trihydroxyphenyl)but-3-en-2-one **19**

Yield: 52 mg (54%), mp, ^1^H NMR (500 MHz, DMSO-*d*_6_): 2.27 (3H, s, C*H_3_*C=O), 6.39 (1H, d, *J* = 16.2 Hz, C*H*C=O), 6.61 (2H, s, Ar-*H*), 7.36 (1H, d, *J* = 16.2 Hz, C*H*Ar), 8.73 (3H, br, OH); ^13^C NMR (62.5 MHz, DMSO-*d*_6_): 27.1 (CH_3_), 107.7 (Ph), 124.0 (*C*HC=O), 124.6 (Ar), 136.5 (Ar), 144.4 (*C*HAr), 146.1 (Ar), 197.6 (C=O). Anal. Calc. for C_10_H_10_O_4_: C 61.85, H 5.19. Found: C 61.79, H 5.13.

### 2.3. Synthesis of qf-Aβ(*1–7*)C

The protocol of qf-Aβ(1–7)C synthesis was modified from a previous protocol [37]. The peptide Aβ(1–7)C (sequence DAEFRHDC, which corresponds to residues 1 to 7 of the Aβ peptide followed by a cysteine residue), was prepared by the Fmoc-polyamide method on a PS3 peptide synthesizer (Rainin Instrument Co., Inc.; Woburn, MA, USA). The crude peptide was purified by HPLC. The thiol-reactive Alexa-350 (Alexa Fluor^®^ 350 C5-maleimide, Molecular Probes/ThermoFisher Scientific, Waltham, MA, USA), and amine-reactive Dabcyl [4-(4′-N, N-dimethylaminophenyl)azobenzoic acid, succinimidyl ester] (Invitrogen, Carlsbad, CA, USA) were used as the fluorescence donor and quencher, respectively. Tris (2-carboxyethyl) phosphine (TCEP) was dissolved in DMSO to make a 50 mM stock solution. One milligram of Alexa-350 in 100 μL of 200 mM MOPS buffer (pH 7.2) and 1 mg of peptide in 850 μL of 200 mM MOPS buffer (pH 7.2) were mixed with 50 μL of TCEP stock solution. The molar ratio of peptide:dye is 1:1.7. The mixture was reacted for two hours in the dark at room temperature with gentle inversion (91 rpm). The dye-labeled peptide was purified by HPLC, identified on a MALDI mass spectrometer, and lyophilised. Alexa-350 labeled Aβ(1–7)C was dissolved in DMSO. The quench dye Dabcyl (7.5 mg) was dissolved in 700 μL DMSO (final concentration 29.3 mM). Alexa-350 labeled Aβ(1–7)C was mixed with Dabcyl to obtain the molar ratio of 1:10 (peptide:Dabcyl). Then, 1/20 volume of N-methylmorpholine (NMM) was added to achieve a mild basic condition. The mixture was reacted with gentle inversion for 2 h at room temperature in the dark. After the reaction, the reaction mixture was centrifuged at 19,000 g at 4 °C for 15 min. The supernatant was collected and the double-labeled peptide in the supernatant, designated qf-Aβ(1–7)C, was purified by HPLC, identified on a MALDI mass spectrometer, lyophilised, and stored at −20 °C.

### 2.4. Synthesis of qf-Aβ(12–16)AAC-EDANS

The protocol of qf-Aβ(12–16)AAC synthesis was modified from a previous protocol [38]. The Aβ(12–16)AAC (sequence VHHQKAAC, which corresponds to residues 12 to 16 of the Aβ peptide followed by two alanine residues and one cysteine residue) was synthesised on a PS3 peptide synthesiser. The thiol-reactive EDANS C2 maleimide was used as the fluorescence donor instead of Alexa-350. Tris (2-carboxyethyl) phosphine (TCEP) was dissolved in DMSO to make a 50 mM stock solution. About 5 mg of crude Aβ(12–16)AAC in 850 μL of 200 mM MOPS buffer (pH 7.2) and 1 mg of EDANS in 100 μL of DMSO were mixed with 50 μL of TCEP stock solution. The molar ratio of peptide:dye is 2:1. The mixture was reacted at room temperature in the dark overnight with gentle inversion (91 rpm). The EDANS-labeled Aβ(12–16)AAC was purified by HPLC and identified on a MALDI mass spectrometer. EDANS-labeled Aβ(12–16)AAC was lyophilised, re-dissolved in DMSO, and mixed with Dabcyl. The molar ratio of peptide and Dabcyl is 1:10. To the mixture, 1/20 volume NMM was added in and reacted with gentle inversion for 2 h at room temperature in dark. After the reaction, the reaction mixture was centrifuged at 19,000 g at 4 °C for 15 min. The supernatant was collected and the double-labeled peptide, designated qf-Aβ(12–16)AAC-EDANS, was purified by HPLC, identified on a MALDI mass spectrometer, lyophilised, and stored at −20 °C.

### 2.5. Biological Evaluation

#### 2.5.1. Cell Culture

Human neuroblastoma SH-SY5Y cells were purchased from the American Type Culture Collection (ATCC, USA) and cultured in Dulbecco’s modified Eagle’s medium: Nutrient Mixture F-12 (DMEM/F-12, Life technologies, Gibco BRL, Grand Island, NY, USA) supplemented with 10% fetal bovine serum (FBS; Biological industries, Cromwell, CT, USA) in 5% CO_2_ at 37 °C.

#### 2.5.2. Neprilysin Aβ-Degrading Activity Assay Using qf-Aβ(1–7)C or qf-Aβ(12–16)AAC-EDANS as Substrate

To determine the optimal cell density for SH-SY5Y cell, 200 µL of SH-SY5Y cells with 2.5 × 10^4^ cells/mL cell density were seeded in 96-well plate and incubated for 24 h before replacing the medium by DMEM-F12 medium without 10% FBS supplement with or without the compounds (final concentration 5 µM). The cells were then incubated for 24 h followed by the replacement of medium by 200 µL of assay buffer (5.5 mM D-glucose, 0.3 mM sodium pyruvate, 25 mM sodium bicarbonate, 1.5 μM Zinc sulfate) containing 2 µM qf-Aβ(1–7)C, incubated for 1.5 h or 4 µM qf-Aβ(12–16)AAC-EDANS, incubated for 1 h, respectively. A quantity of 150 µL of reacted assay buffer was transferred to another 96-well plate (costar, black plate) for fluorescence measurement on Infinite M1000 pro (Tecan). The excitation wavelength was set at 346 nm and the emission wavelength at 442 nm for qf-Aβ(1–7)C. The excitation wavelength was set at 355 nm and the emission wavelength at 500 nm for qf-Aβ(12–16)AAC-EDANS. For the thiorphan inhibition assay, after removing the compound-containing medium, 50 µL of thiorphan in the assay buffer (final concentration 50 μM) was added and incubated for 30 min. Then, 150 µL of the assay buffer containing qf-Aβ(1–7)C or qf-Aβ(12–16)AAC-EDANS was added. The final peptide concentration is 2 µM for qf-Aβ(1–7)C and 4 µM for qf-Aβ(12–16)AAC-EDANS. The following incubation time for peptide digestion is 1.5 h for qf-Aβ(1–7)C and 2 h for qf-Aβ(12–16)AAC-EDANS.

### 2.6. Statistical Analysis

All the experiments were repeated three times and, each time, the process was performed in triplicate. The average intensity of three experiments was normalised to the control group. All statistical analyses were performed using GraphPad Prism 8 software (Graphpad). Statistical significances were compared with the control group and determined by one-way ANOVA with Dunnett’s multiple comparisons test (*, *p* < 0.05; **, *p* < 0.01; ***, *p* < 0.001; ****, *p* < 0.0001). Data are expressed as mean value ± SD.

Interventionary studies involving animals or humans, and other studies that require ethical approval, must list the authority that provided approval and the corresponding ethical approval code.

## 3. Results and Discussion

### 3.1. Design

**Figure 2:**
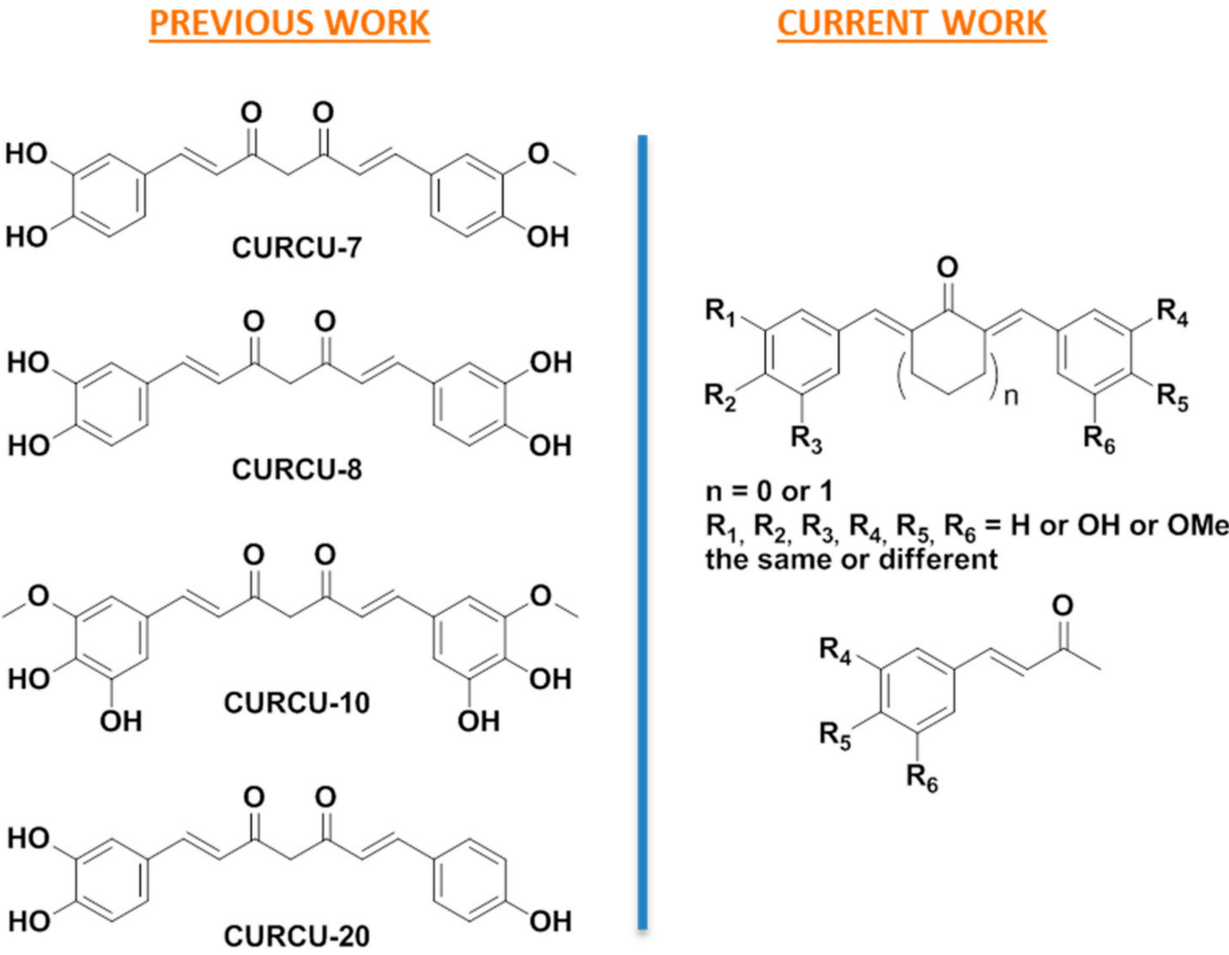
The schematic representation of the design of this work based on previous reports [25].

As part of an effort to develop improved curcuminoids, the design of monocarbonyl analogs of curcumin (MACs) as a highly attractive alternative class of compounds was chosen [27,28,29]. MACs usually demonstrate higher potency than the diketo-curcuminoid counterparts, they are more readily produced, more stable, and they exhibit improved pharmacological profiles [39,40]. Based on the above, a small library of symmetric and unsymmetric hydroxylated monocarbonyl curcumin derivatives was designed using acetone or cyclohexanone as linkers between the aromatic moieties (Figure 2). Some of these compounds are indeed the monocarbonyl analogues of the most active diketo curcuminoids previously reported [25].

### 3.2. Synthesis

**Scheme 1:**
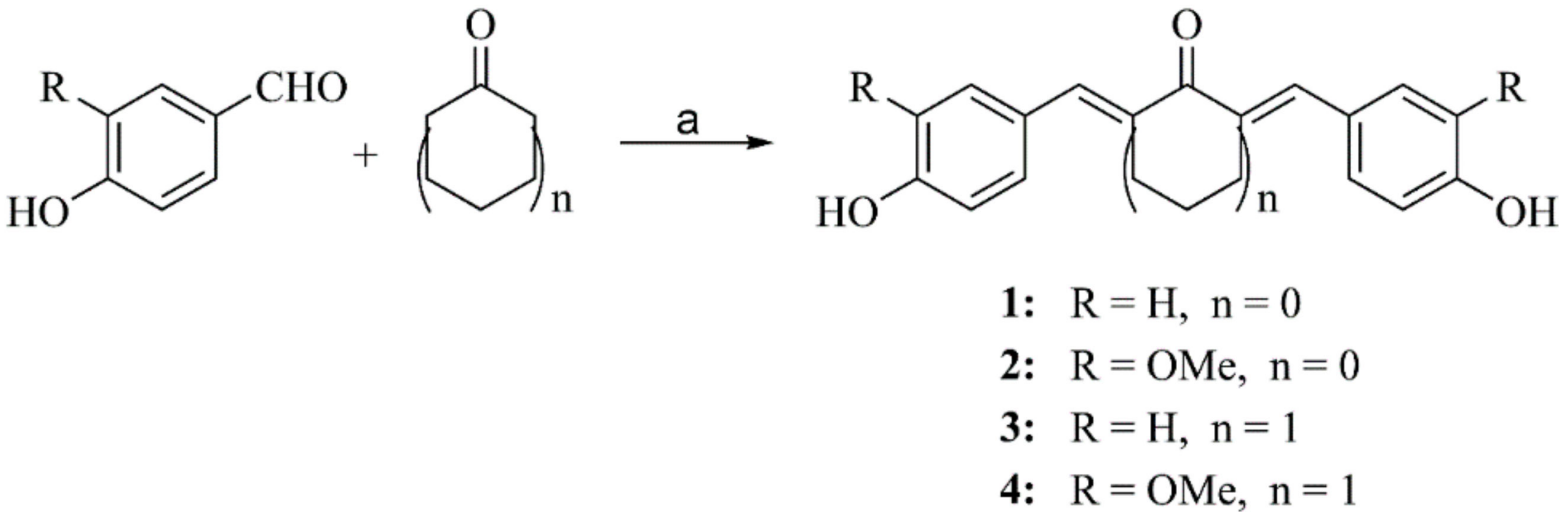
Synthetic route to symmetric compounds **1**–**4**. Reagents and conditions: (a) conc. HCl, EtOH, rt, 24 h (66–73%).

The target compounds were synthesized following five different routes depending on their structure. Thus, symmetric compounds **1**–**4** were prepared by a conventional Claisen–Schmidt reaction from the corresponding aromatic aldehydes and acetone or cyclohexanone, by using a catalytic amount of concentrated hydrochloric acid (Scheme 1).

**Scheme 2:**
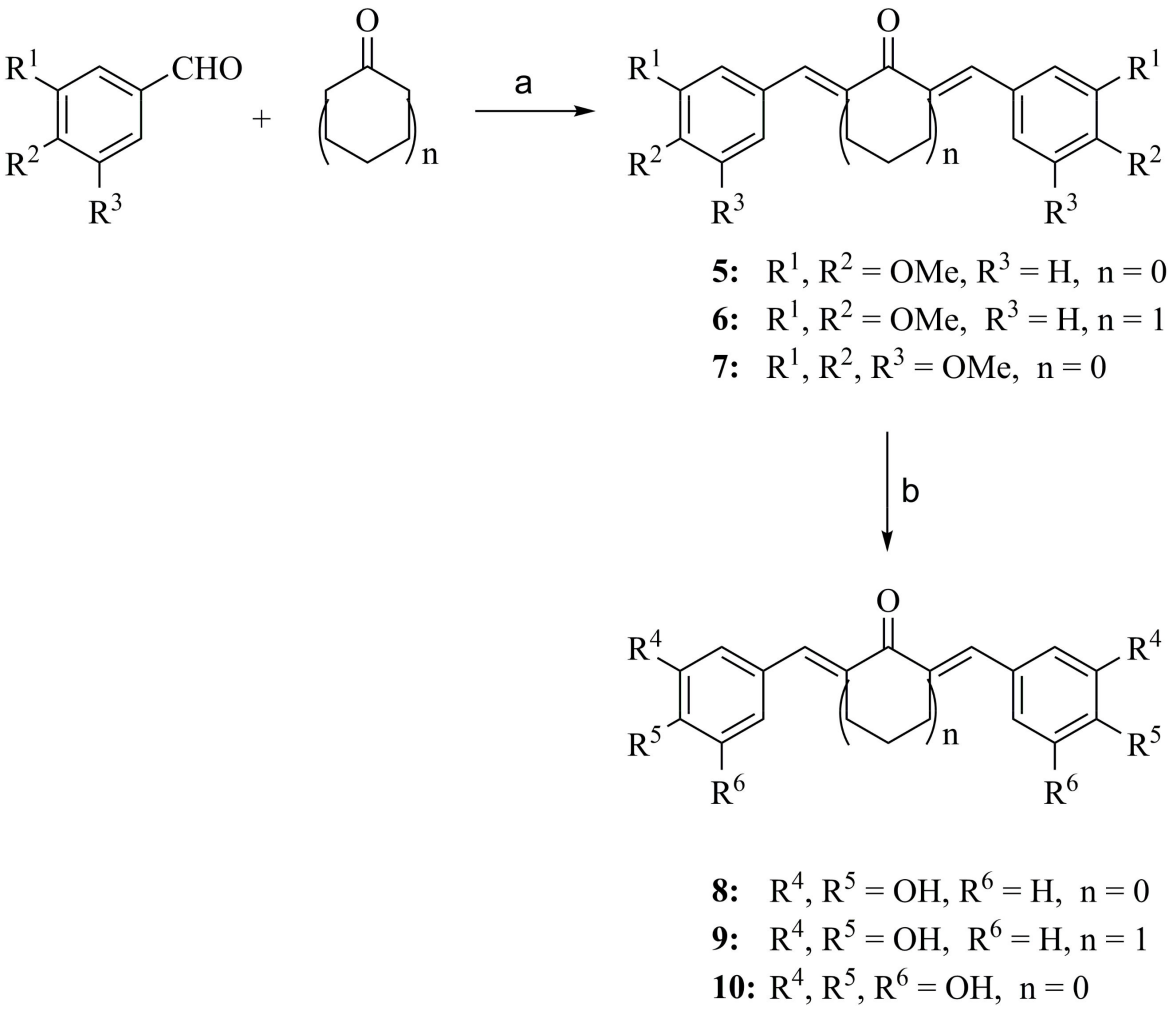
Synthetic route to polyhydroxylated compounds **8**–**10** via the methoxy-protected intermediates **5**–**7**. Reagents and conditions: (a) NaOH, EtOH, rt, 2 h (69–72%); (b) BBr_3_, DCM (dry), −20 °C to rt, 3 h (45–71%).

In a similar approach, symmetric compounds **8**–**10** were synthesized from the methoxy-protected intermediates **5**–**7**, prepared from the corresponding aromatic aldehydes and acetone or cyclohexanone, in alkaline conditions (Scheme 2) [28]. It is worth noting that the Claisen–Schmidt condensation of the unprotected aldehyde coupling partners did not proceed well in these conditions.

However, the commercial availability of the starting materials, and the deprotection of the intermediates **5**–**7**, following a boron tribromide protocol, ensured an efficient two-step route to the target compounds **8**–**10**.

**Scheme 3:**
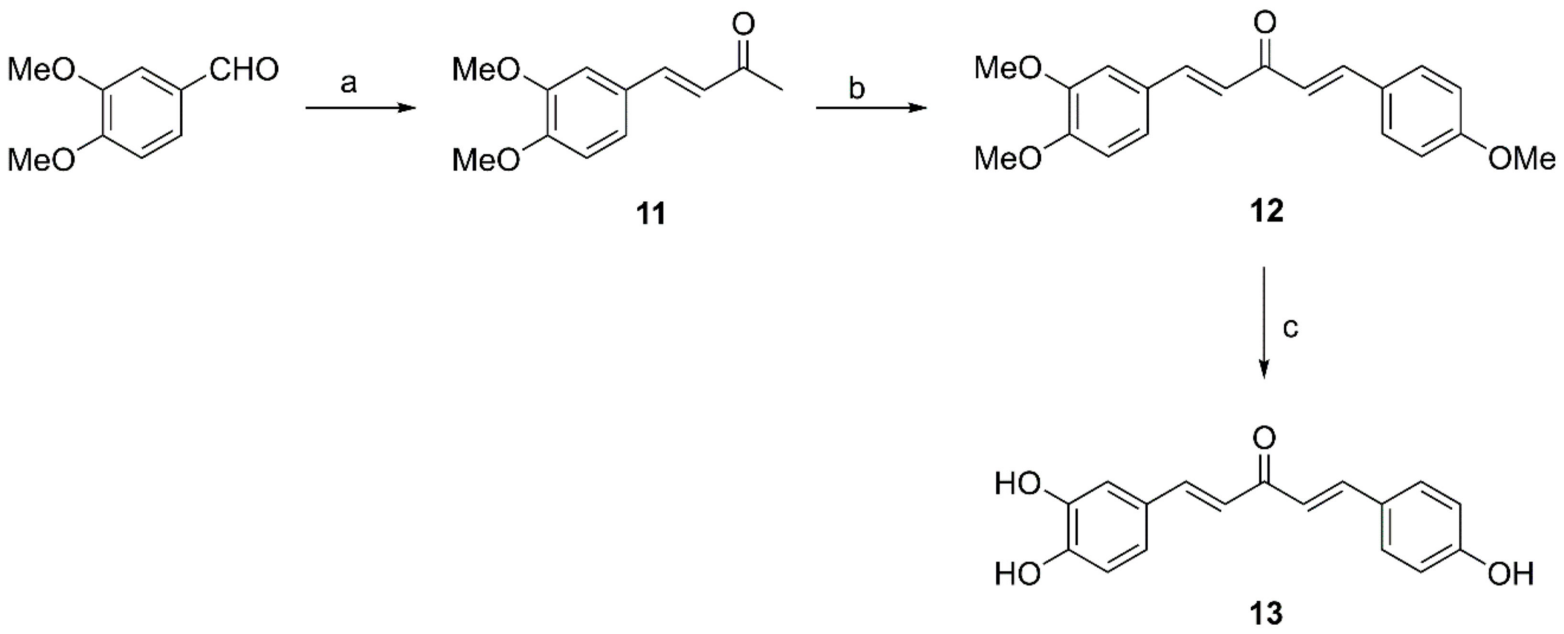
Synthetic route to unsymmetric compound **13**. Reagents and conditions: (a) acetone, NaOH 10%, rt, 16 h (55%); (b) *p*-anisaldehyde, NaOH, EtOH, H_2_O, rt, 16 h (35%); (c) BBr_3_, DCM (dry), −20 °C to rt, 3 h (34%).

Synthesis of the unsymmetrical compound **13** proceeded via the “half” derivative **11,** prepared from veratraldehyde, using a large excess of acetone, in order to achieve mono-derivatization at this step (Scheme 3). Further condensation of **11** with p-anisaldehyde afforded the protected unsymmetric intermediate **12**, following a slightly modified Claisen–Schmidt in comparison to the protocol used for **5**–**7**. Treatment of **12** with boron tribromide, afforded the target compound **13,** in 65.4% overall yield from the starting aldehyde.

**Scheme 4:**
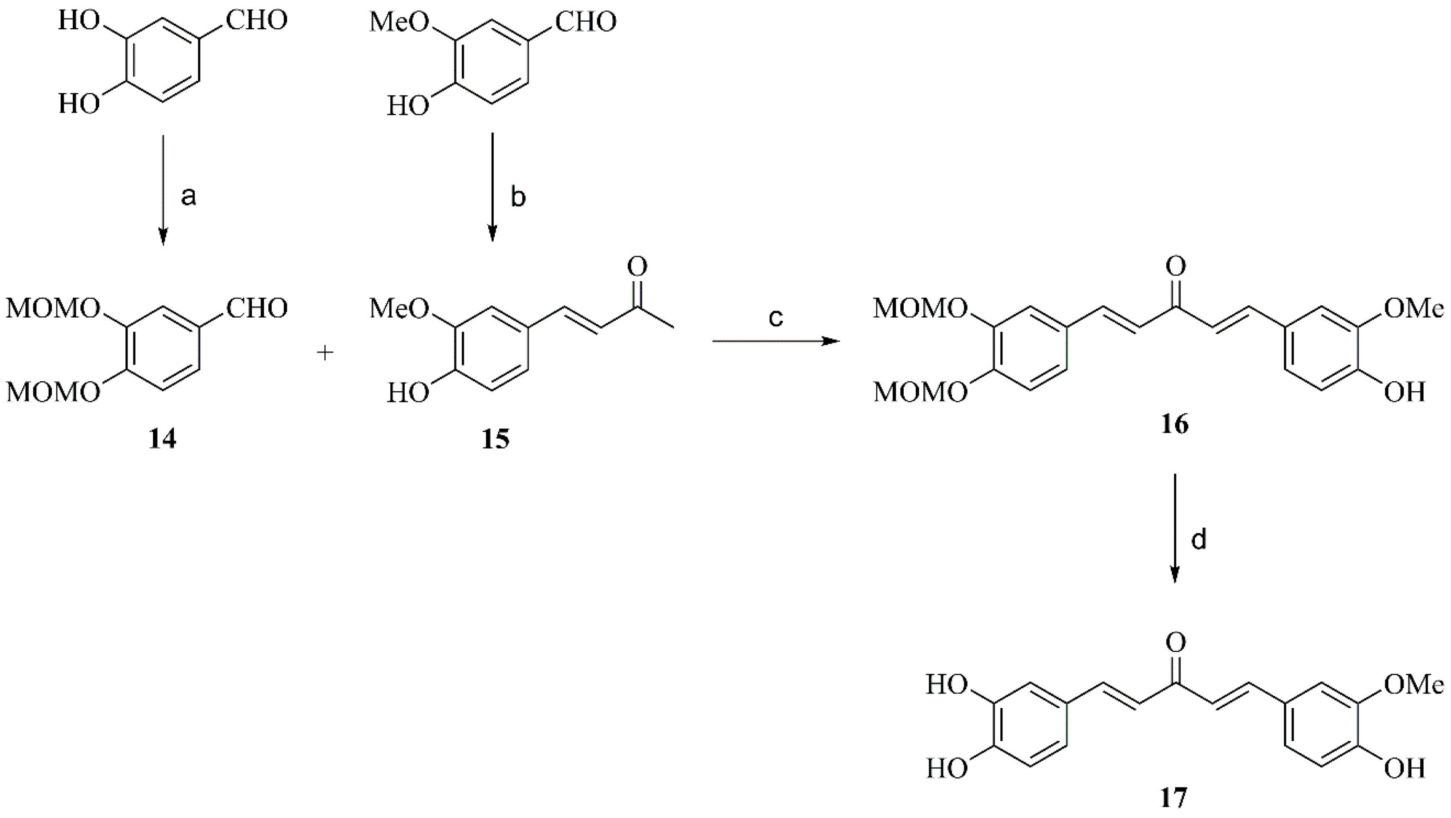
Synthetic route to unsymmetric compound **17** using the MOM-protective group protocol. Reagents and conditions: (a) CH_3_OCH_2_Cl, K_2_CO_3_, DMF (dry), rt, 16 h (83%); (b) 1. acetone, NaOH 10% 16 h, rt, 2. HCl 1M, rt (85%); (c) NaOH, EtOH, H_2_O, rt, 16 h (45%); (d) HCl 3M, MeOH, 65 °C (45%).

For the synthesis of the unsymmetrical compound **17**, which bears hydroxyl groups and a methoxy group, an orthogonal protection strategy was required (Scheme 4). Therefore, starting from the commercially available 3,4-dihydroxybenzaldehyde and vanillin, we first prepared the MOM-protected aldehyde **14** and ketone **15**, respectively. The latter compounds were condensed via a Claisen–Schmidt reaction to afford the partially MOM-protected intermediate **16**, which was selectively deprotected under acidic conditions to afford the target compound **17**.

**Scheme 5:**
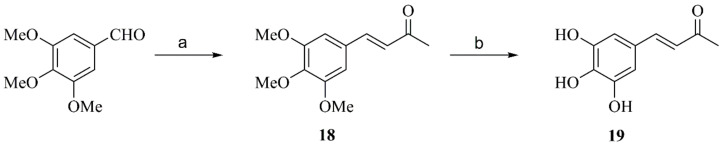
Synthetic route to “half” trihydroxy compound **19** via the trimethoxy intermediate **17**. Reagents and conditions: (a) acetone, NaOH 1M, rt, 16 h (44%); (b) BBr_3_, DCM (dry), −20 °C to rt, 3 h (54%).

Finally, the target compound **19** was prepared from 3,4,5-trimethoxybenzaldehyde, after condensation with acetone to afford the “half” derivative **18**, which was subsequently deprotected by using boron tribromide (Scheme 5).

The structures and the purities of the novel or active final compounds were confirmed by ^1^H and ^13^C NMR experiments along with elemental analysis. The structures of the known final and intermediate compounds were confirmed by ^1^H NMR spectroscopy and comparison with data reported in the literature. The ^1^H NMR spectrum of all tested compounds remained the same for at least a month in the stock DMSO solution. No alterations were observed after incubating representative compounds with cell culture medium for 24 h.

### 3.3. Biological Evaluation

#### 3.3.1. Synthesis and Specificity of Fluorescent Peptide NEP Substrates

The effect of the hydroxyl-bearing monocarbonyl compounds on the neprilysin Aβ-degrading activity was evaluated by means of a fluorescence-based Aβ digestion assay. For this purpose, the qf-Aβ(1–7)C and the qf-Aβ(12–16)AAC-EDANS peptides were successfully synthesized based on previous work with slight modifications to increase yield [37,38]. These peptide substrates are designed to carry both a fluorescence reporter and a quencher onto them. In the case of Aβ(1–7)C, Alexa-350 was used as a fluorescent moiety whereas this was replaced by a more economical and chemically attractive fluorescent reporter, the 5-[(2-Aminoethyl)amino]naphthalene-1-sulfonyl (EDANS), in the case of qf-Aβ(12–16)AAC substrate. Both fluorescent peptides were conjugated to Dabcyl as the quencher. Among the Aβ-degrading enzymes including NEP, ECE-1, ACE, IDE, plasmin, NMP-3 and NMP-9, qf-Aβ(1–7)C is sensitive to NEP and IDE (insulin-degrading enzyme) degradation only [37]. On the other hand, qf-Aβ(12–16)AAC is more susceptible to NEP and ACE (angiotensin converting enzyme) [38]. Based on our previous experience, it was initially important to assure that NEP is still able to effectively cleave qf-Aβ(12–16)AAC-EDANS and its selectivity has not been altered by changing the fluorophore.

**Figure 3:**
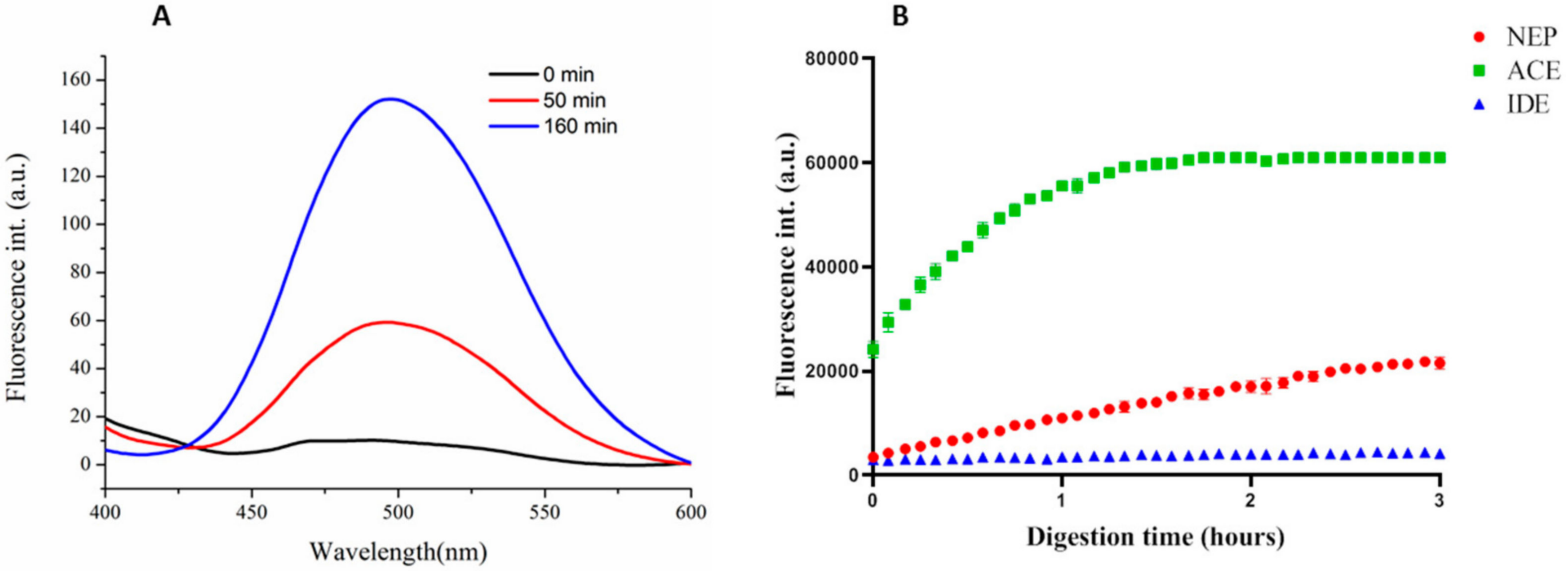
(**A**) The fluorescence spectra of qf-Aβ(12–16)AAC-EDANS digested by 2 nM NEP. qf-Aβ(12–16)AAC-EDANS (4 μM) was digested by 2 nM NEP in 50 mM Tris, 25 mM NaCl, 5 μM ZnCl_2_, pH 7.5 at 37 °C. The fluorescence spectra were recorded at the indicated times with excitation wavelength of 336 nm on spectrofluorometer FP-750 (Jasco). (**B**) Degradation kinetics of qf-Aβ(12–16)AAC-EDANS by NEP, ACE, or IDE. qf-Aβ(12–16)AAC-EDANS (4 μM) was digested by 2 nM NEP, ACE, or IDE in the assay buffer (5.5 mM D-glucose, 0.3 mM sodium pyruvate, 25 mM sodium bicarbonate, 1.5 μM Zinc sulfate) incubated in a 96-well plate at room temperature. The fluorescence intensity at 500 nm was recorded on Infinite M1000 pro (Tecan) every 5 min with excitation at 355 nm. The data are averaged from triplicate samples. The error bar is too small to be seen.

The ability of NEP to degrade the newly prepared peptide qf-Aβ(12–16)AAC-EDANS was evaluated by means of fluorescence spectroscopy. A minute amount of NEP (2 nM) was used in the appropriate buffer and then, upon incubation with the peptide for various time intervals, the fluorescent spectrum was recorded (Figure 3A). There is a significant increase in the fluorescence intensity observed with longer incubation time, which clearly indicates the effective and desirable NEP-assisted cleavage of the peptide. It should be mentioned that no fluorescence was recorded in the absence of NEP, which means that the peptide was completely stable in the buffer used and that the recorded increasing intensity was exclusively the result of Aβ(12–16)AAC cleavage. Furthermore, qf-Aβ(12–16)AAC-EDANS was equally challenged by the same amount of NEP, IDE and ACE to evaluate the substrate specificity. The results presented in Figure 3B demonstrate that qf-Aβ(12–16)AAC-EDANS retains its selectivity as a substrate to ACE and NEP, as was also the case for qf-Aβ(12–16)AAC, which was used in previous works in a comparable way [38].

#### 3.3.2. NEP Aβ-Degrading Activity in Cells and Inhibition Studies

**Figure 4:**
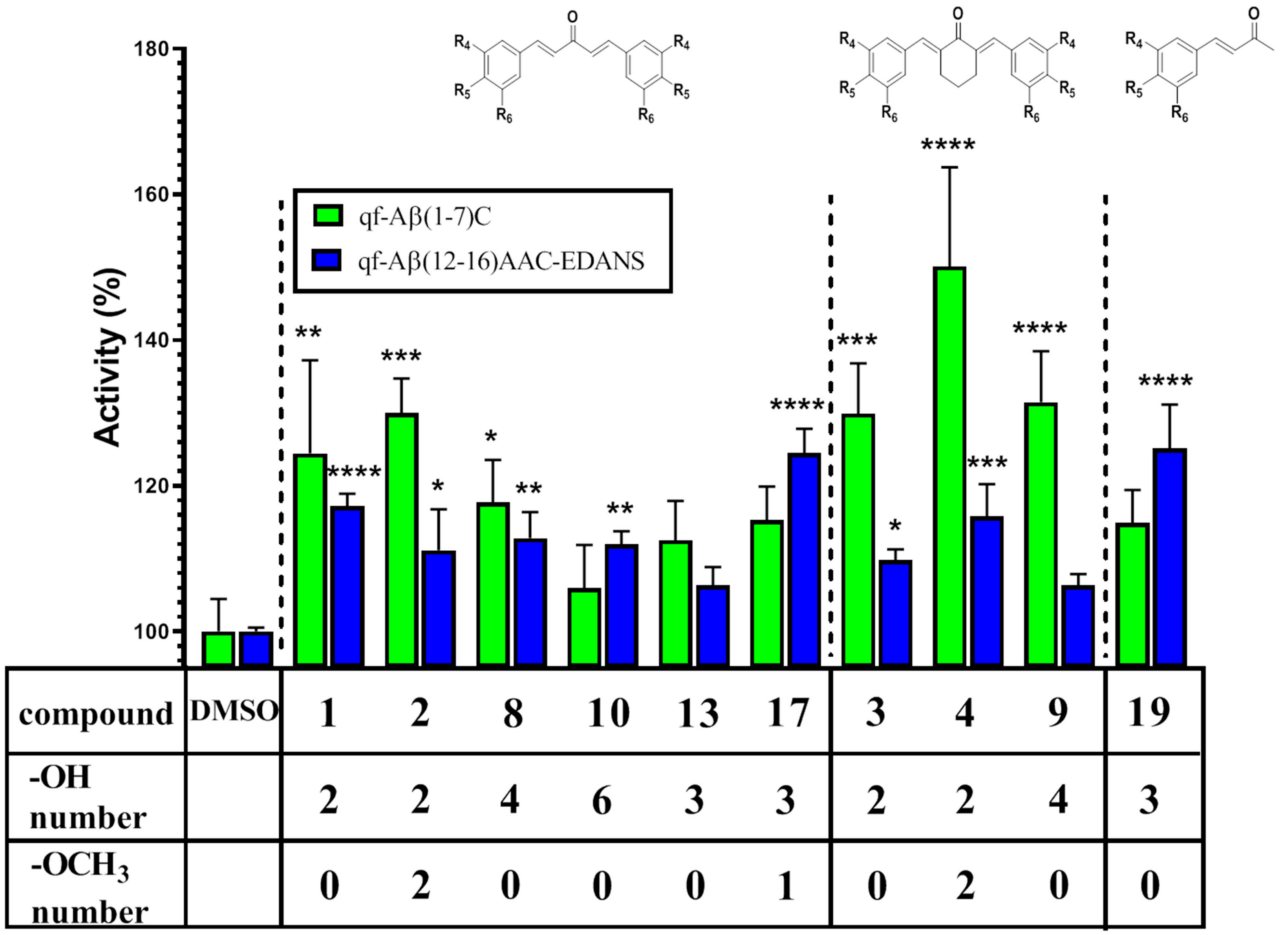
A*β*-degrading activity assay in SH-SY5Y cells incubated for 24 h using qf-A*β*(1–7)C or qf-A*β*(12–16)AAC-EDANS as substrate with 0.5% DMSO (control) or in the presence of the tested compounds (5 μM). The data are the mean ± SD for three independent samples. * *p* < 0.05; ** *p* < 0.01; *** *p* < 0.001; ****, *p* < 0.0001 compared to the control by one-way ANOVA.

Subsequently, the Aβ-degrading activity in SH-SY5Y cells was evaluated by using either qf-Aβ(1–7)C or qf-Aβ(12–16)AAC-EDANS as substrate with or without the tested compounds. The cell viability of SH-SY5Y cells was not affected by the compounds at the 5 μM concentration that was used to assess NEP activity. The results presented in Table 1 and Figure 4 show that compounds **1**, **2**, **3**, **4**, and **8** exhibited a significant fluorescence increase in both assays, whereas compounds **9**, **10**, **17** and **19** exhibited a statistically significant increase in one of the two assays. More specifically, compound **4** was the most active in the qf-Aβ(1–7)C assay whereas derivatives **17** and **19** were of the highest activity in the qf-Aβ(12–16)AAC-EDANS assay. Furthermore, most of the compounds had higher activity increases in the qf-Aβ(1–7)C assay than the qf-Aβ(12–16)AAC-EDANS, except for compounds **10**, **17**, and **19**. These results may be influenced by the fact that NEP is not the only catabolizing enzyme involved; qf-Aβ(1–7)C may also be successfully cleaved by IDE [37], and qf-Aβ(12–16)AAC-EDANS is, similarly, a good substrate for ACE [38]. Thiorphan, a NEP-specific inhibitor was, therefore, used to selectively block the activity of NEP and record any remaining fluorescent intensity presumably resulting from IDE or ACE cleavage, respectively.

**Figure 5:**
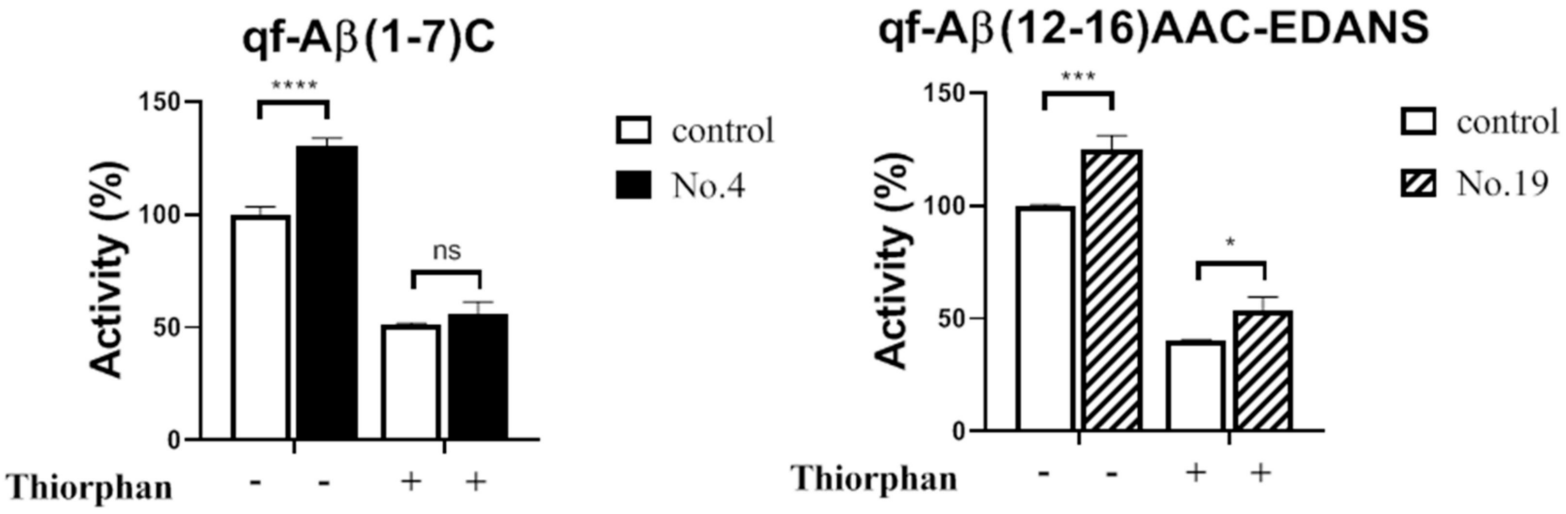
Thiorphan inhibition assay. SH-SY5Y cells were incubated for 12 h in the presence or not of compound **4** or **19** (5 μM), followed by thiorphan (50 μM) for 30 min before Aβ-degrading activity was measured using the appropriate substrate. The data are the mean ± SD for three independent samples. * *p* < 0.05; *** *p* < 0.001; ****, *p* < 0.0001 compared to the control by one-way ANOVA.

After incubating SH-SY5Y cells with or without compound **4**, the cells were treated with 50 μM thiorphan prior to addition of qf-Aβ(1–7)C. The same experimental procedure was followed with compound **19** and qf-Aβ(12–16)AAC-EDANS. In both cases, thiorphan was found to significantly decrease substrate cleavage activity to more than 50% of the untreated control, as shown in Figure 5. More specifically, in the case of qf-Aβ(1–7)C substrate, thiorphan reduced the compound **4** related fluorescence increase by nearly 70% and there was no statistically significant difference compared to the control. This result strongly demonstrates that the increased activity induced by compounds **4** was almost exclusively due to its effect on NEP. Interestingly, the thiorphan treatment resulted in a small residual fluorescence compared to the control when qf-Aβ(12–16)AAC-EDANS substrate was used, in the presence or absence of compound **19**. This observation not only indicates that the fluorescence increase caused by compound **19** may be related to NEP activation but also, to some extent, that ACE activation may be involved.

With regard to the qf-Aβ(1–7)C assay, it is noteworthy that for the mono- or di-substituted symmetric compounds **1**–**9**, the cyclohexanone derivatives were found to be more active than the corresponding acetone derivatives. In addition, derivative **2** exhibited higher activity than its diketo counterpart; specifically, curcumin and compound **8** was only slightly less active than the di-hydroxylated bisdemethoxycurcumin [25]. Moreover, their cyclohexanone analogues **4** and **9** were found to increase NEP activity even more. On the other hand, the tri-substituted derivatives **10** and **19**, and the unsymmetrical **13** and **17**, resulted in a non-statistically significant increase in activity compared to the control. Interestingly, the activity increase did not seem to be related to the number of hydroxyl groups. In fact, compound **10**, which has 6 hydroxyl groups, did not cause a significant activity increase in the qf-Aβ(1–7)C assay and exhibited the lowest % increase in all tested compounds. Consequently, the results of this work do not seem to provide further support to previous observations on diketo curcuminoids, where at least three hydroxyl groups were required for neprilysin upregulation [25]. Collectively, the two sets of results indicate that it is not only the degree of hydroxylation that is the main predominant factor affecting the potential NEP activity enhancement. In the case of diketo curcuminoids, the enhanced solubility of the polyhydroxylated derivatives may have provided the activity benefit observed. However, for the monocarbonyl analogues, which are known to have superior aqueous solubility and stability compared to their diketo counterparts [40,41], the activity was affected in a different way. Our results suggest that balancing the phenolic character with vanillin-based aromatic motif and the more rigid and bulky cyclohexanone moiety resulted in the highest activity for compound **4** in the qf-Aβ(1–7)C assay. It is also worth mentioning that the ease of preparation and purification, as well as the lower synthetic cost, of this compound in multigram scale from simple starting materials, makes it a highly desirable pharmaceutical candidate.

Our results demonstrate a very highly promising role of the monocarbonyl curcumin derivatives as proteolytic potentiation agents via NEP. From a mechanistic point of view, the increased proteolysis has been previously suggested to be related to promoted expression, secretion and (allosterically) catalytic activation of the targeted endopeptidase as well as epigenetic regulation at the level of histones [20]. More specifically, the histone deacetylase (HDAC) inhibitors, such as valproic acid and trichostatin, have been related to reactivated NEP gene expression via blocking of the competitive binding of HDACs to the NEP promoter [17]. Curcumin and a small library of diketone curcumin analogues have been shown to act as promising HDAC inhibitors [42]. Furthermore, PPARγ agonists have also been shown to induce Aβ degradation by increasing apolipoprotein E (ApoE), which has been shown to cause an increase in the concentration of NEP [43,44]. To this end, curcumin may act as a potent PPAR gamma agonist resulting in the inhibition of Abeta-induced inflammation in astrocytes [45]. In addition, NEP activity has been found to be reduced in the presence of Aβ-generated reactive oxygen species (ROS) [46]. In that respect, the work of Zheng et al. reported superior anti-oxidant activity for monocarbonyl curcumin derivatives, including some of our tested compounds [47]. This may contribute, at least partially, to the NEP activity enhancement observed herein. There is no similar report and comparative study for any of the other NEP regulating pathways described earlier. Finally, there is not a well-defined structure–activity-relationship profile for small molecules able to enhance the catalytic activity (or production) of such proteases. Consequently, further elucidation of the specific mode of action of the successful derivatives is underway.

## 4. Conclusions

Ten hydroxylated monocarbonyl curcumin derivatives were successfully synthesized and evaluated for their potential to enhance the activity of NEP in fluorescence-based Aβ digestion and inhibition assays. The cyclohexanone-bearing vanillin derivative resulted in a highly significant increase in NEP activity. This molecule can be readily prepared at large scale, high purity and low cost. Our extremely encouraging set of results prompts us to further exploit the potential application of this molecule against AD.

## Figures and Tables

**Table 1:** Comparison of Aβ-degrading activity by using qf-Aβ(1–7)C or qf-Aβ(12–16)AAC-EDANS. CURCU-8 is 1,7-Bis(3,4-dihydroxyphenyl)-1,6-heptadiene-3,5-dione. ^a^ Data retrieved from the previous report [25].

Compound	% Activity	
	qf-Aβ(1–7)C	qf-Aβ(12–16)AAC-EDANS
Control	100 ± 4.47	100 ± 0.5
1	124.45 ± 12.78	117.17 ± 1.73
2	129.96 ± 4.76	111.13 ± 5.61
3	129.88 ± 6.92	109.78 ± 1.49
4	150.08 ± 13.63	115.78 ± 4.43
8	117.70 ± 5.85	112.78 ± 3.57
9	131.46 ± 7.02	106.35 ± 1.53
10	105.95 ± 5.91	111.92 ± 1.81
13	112.49 ± 5.41	106.30 ± 2.54
17	115.29 ± 4.60	124.48 ± 3.34
19	114.86 ± 4.57	125.10 ± 6.02
CURCU-8	126.10 ± 5.39 ^a^	125.134 ± 8.22

## Data Availability

The data presented in this study are available in this article and Supplementary Materials.

## Supplementary Materials

The following are available online at https://www.mdpi.com/article/10.3390/biomedicines9080955/s1, Tables S1-S22 ^1^H, ^13^C and 2D NMR spectra.
