# Supplementary material for: Synthesis and Biological Evaluation of Hydroxylated Monocarbonyl Curcumin Derivatives as Potential Inducers of Neprilysin Activity
Source: Biomedicines. 2021 Aug 3;9(8):955. doi: 10.3390/biomedicines9080955 (PMC8394082; doi:10.3390/biomedicines9080955)
Supplement: Supplementary file 1 [file biomedicines-09-00955-s001.zip › biomedicines-1286140 supplementary.pdf]

Synthesis and Biological Evaluation of Hydroxylated Monocarbonyl Curcumin Derivatives as Potential Inducers of Neprilysin Activity

Dimitris Matiadis[1], See-Ting Ng [2], Eric H.-L. Chen [2], Georgia Nigianni [1,3], Veroniki P. Vidali [3], Aleksander Canko [3], Rita P.-Y. Chen [2,4,5,*] and Marina Sagnou [1,*]

[1]NCSR "Demokritos", Institute of Biosciences & Applications, Patr. Grigoriou & Neapoleos 27, Athens, 153 41, Greece

[2]Institute of Biological Chemistry, Academia Sinica, No. 128, Sec. 2, Academia Rd., Nankang, Taipei 115, Taiwan

[3]NCSR "Demokritos", Institute of Nanoscience & Nanotechnology, Patr. Grigoriou & Neapoleos 27, Athens, 153 41, Greece

[4]Institute of Biochemical Sciences, National Taiwan University, No. 1, Sec. 4, Roosevelt Rd., Taipei 106, Taiwan

[5]Neuroscience Program of Academia Sinica No. 128, Sec. 2, Academia Rd., Nankang, Taipei 115, Taiwan

Supporting Information

Contents

NMR spectra 2

NMR spectra

(1E,4E)-1,5-bis(4-hydroxyphenyl)penta-1,4-dien-3-one **1**

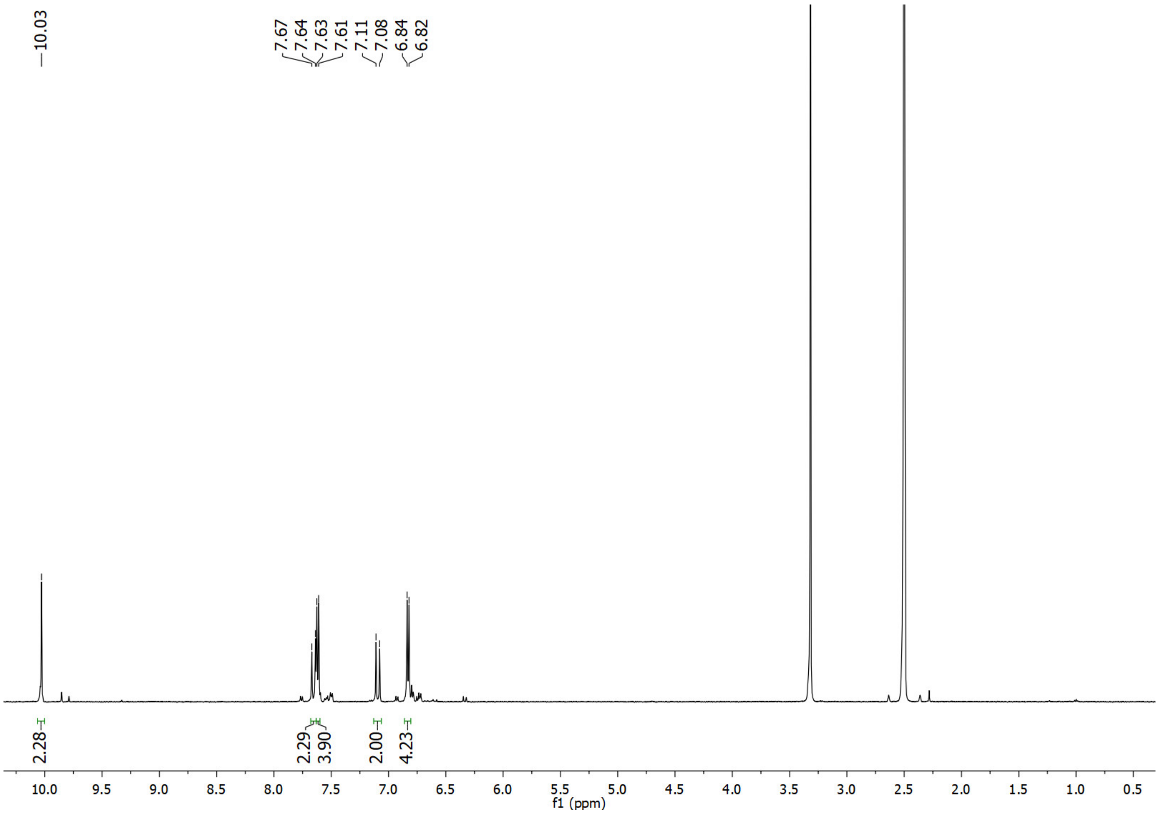

Figure S1. ^{1}H NMR spectrum (500 MHz, DMSO-d_6, δ ppm)

(1E,4E)-1,5-bis(4-hydroxy-3-methoxyphenyl)penta-1,4-dien-3-one **2**

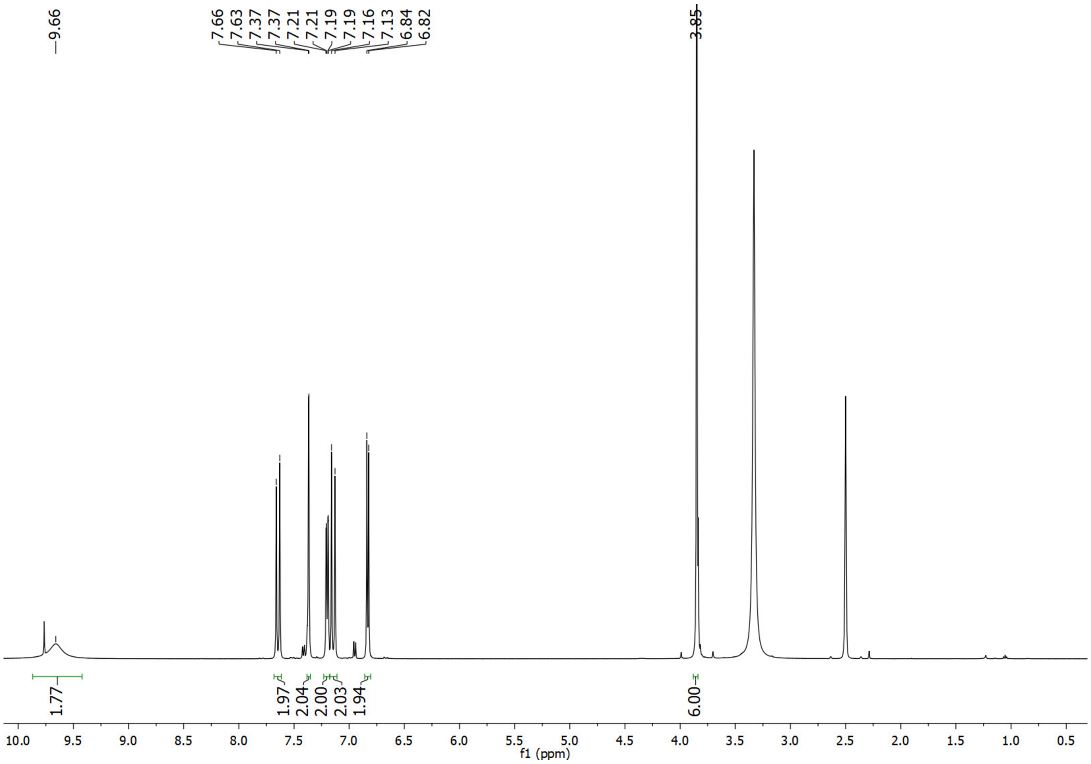

Figure S2. ^{1}H NMR spectrum (500 MHz, DMSO-d_6, δ ppm)

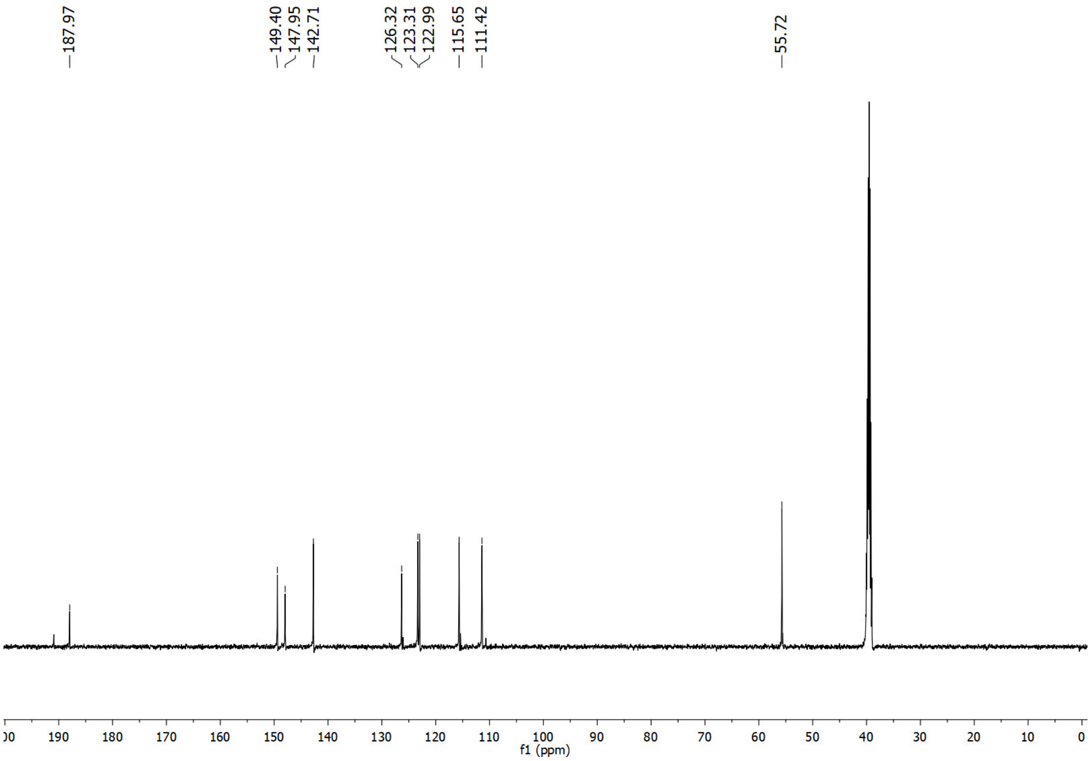

Figure S3. ^{13}**C NMR spectrum** (125 MHz, DMSO-d_6, δ ppm)

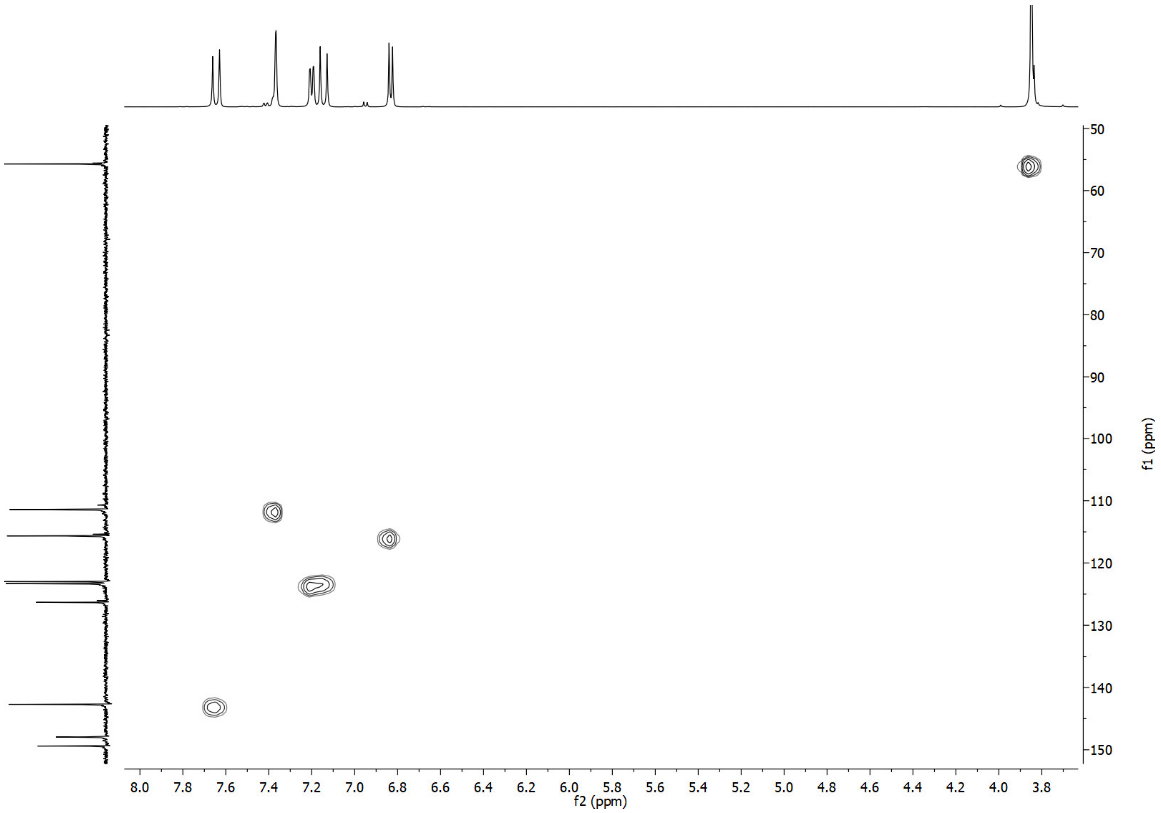

Figure S4. ^{1}**H-**^{13}**C HSQC spectrum**

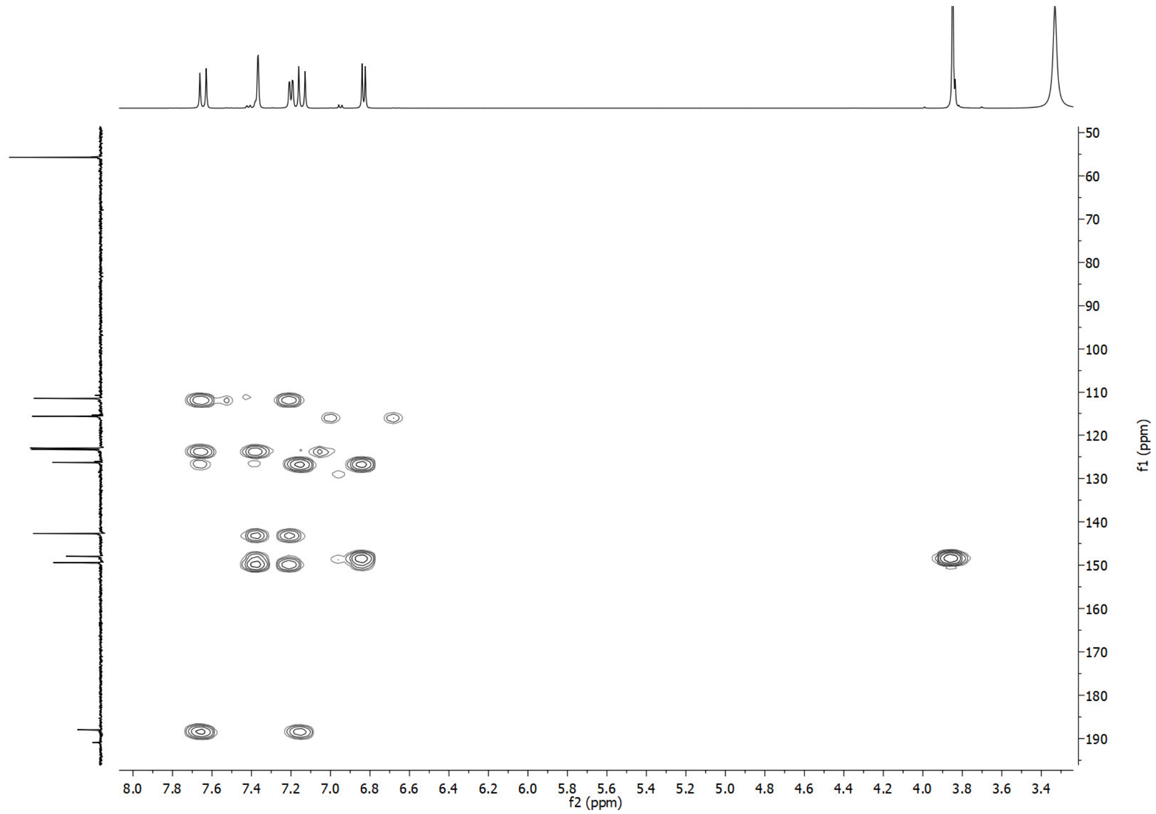

Figure S5. ^{1}H-^{13}C HMBC spectrum

2,6-bis((E)-4-hydroxybenzylidene)cyclohexan-1-one **3**

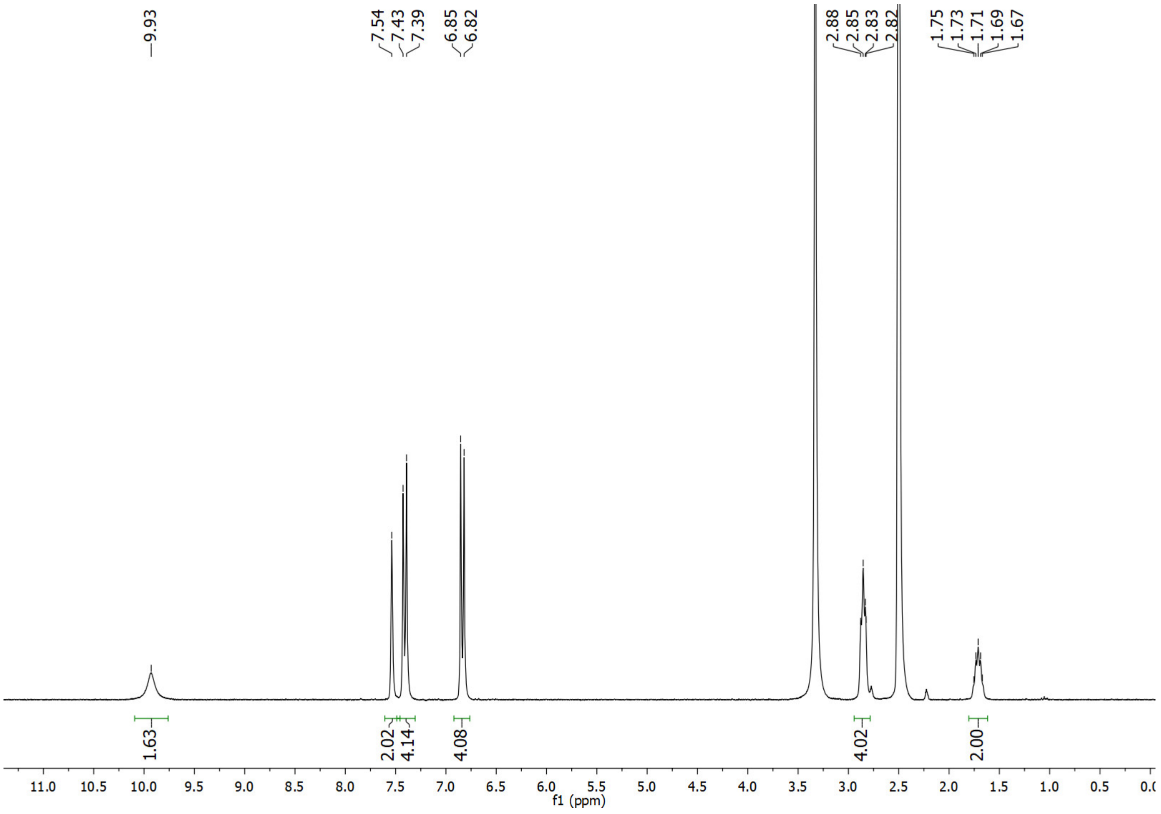

Figure S6. ^{1}H NMR spectrum (500 MHz, DMSO-d_6, δ ppm)

2,6-bis((E)-4-hydroxy-3-methoxybenzylidene)cyclohexan-1-one **4**

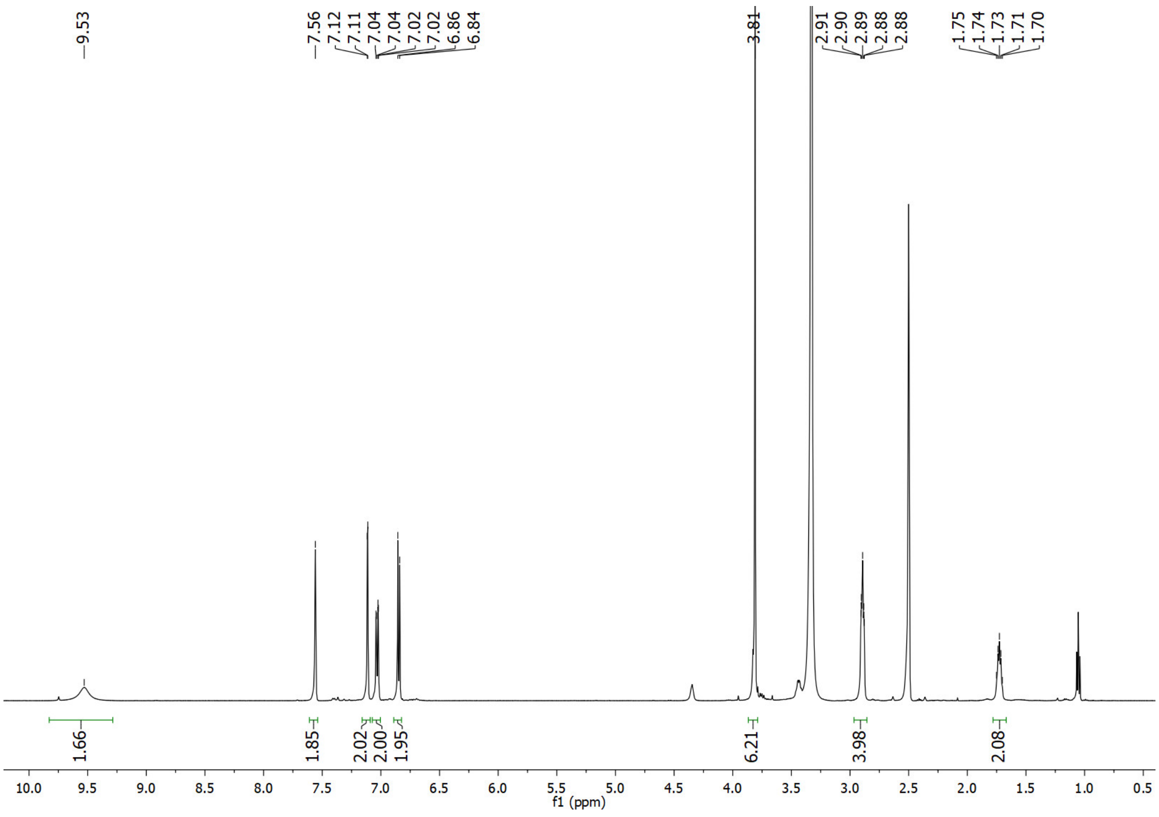

Figure S7. ^{1}H NMR spectrum (500 MHz, DMSO-d_6, δ ppm)

(1E,4E)-1,5-bis(3,4-dihydroxyphenyl)penta-1,4-dien-3-one **8**

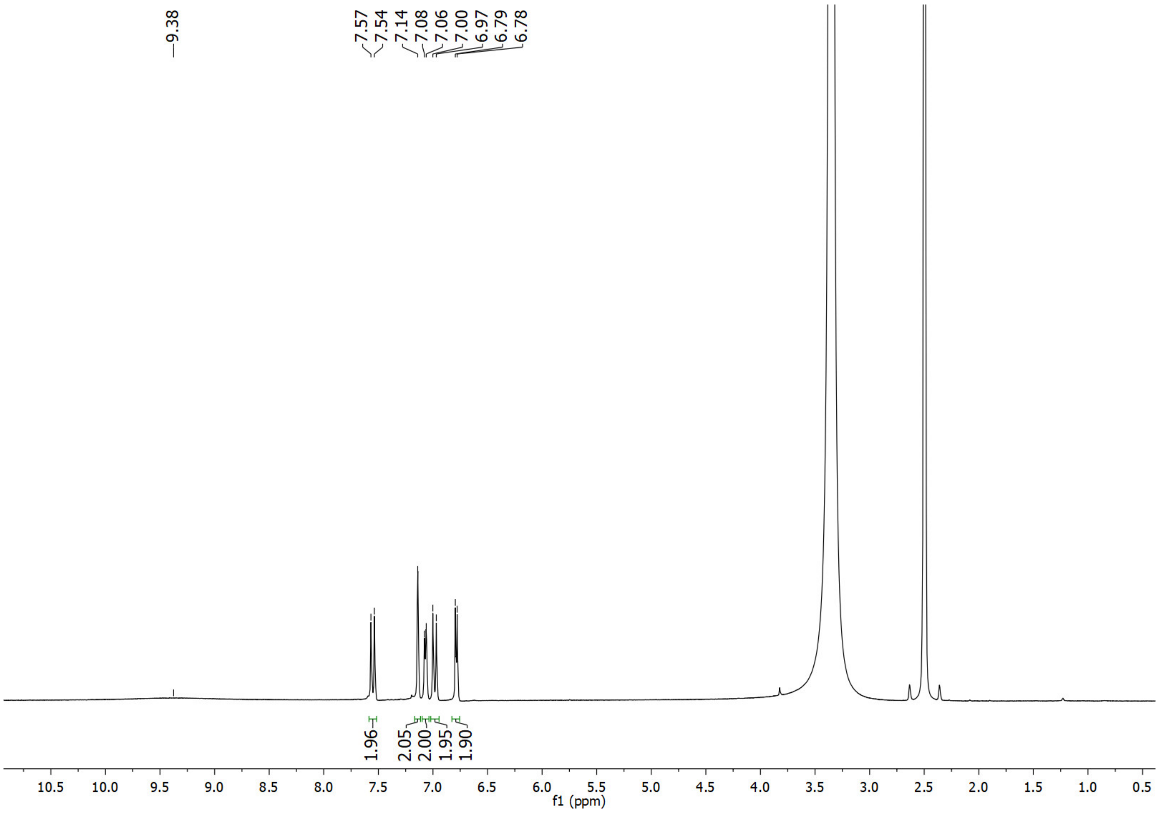

Figure S8. ¹H NMR spectrum (500 MHz, DMSO-d_6, δ ppm)

(2E,6E)-2,6-bis(3,4-dihydroxybenzylidene)cyclohexanone **9**

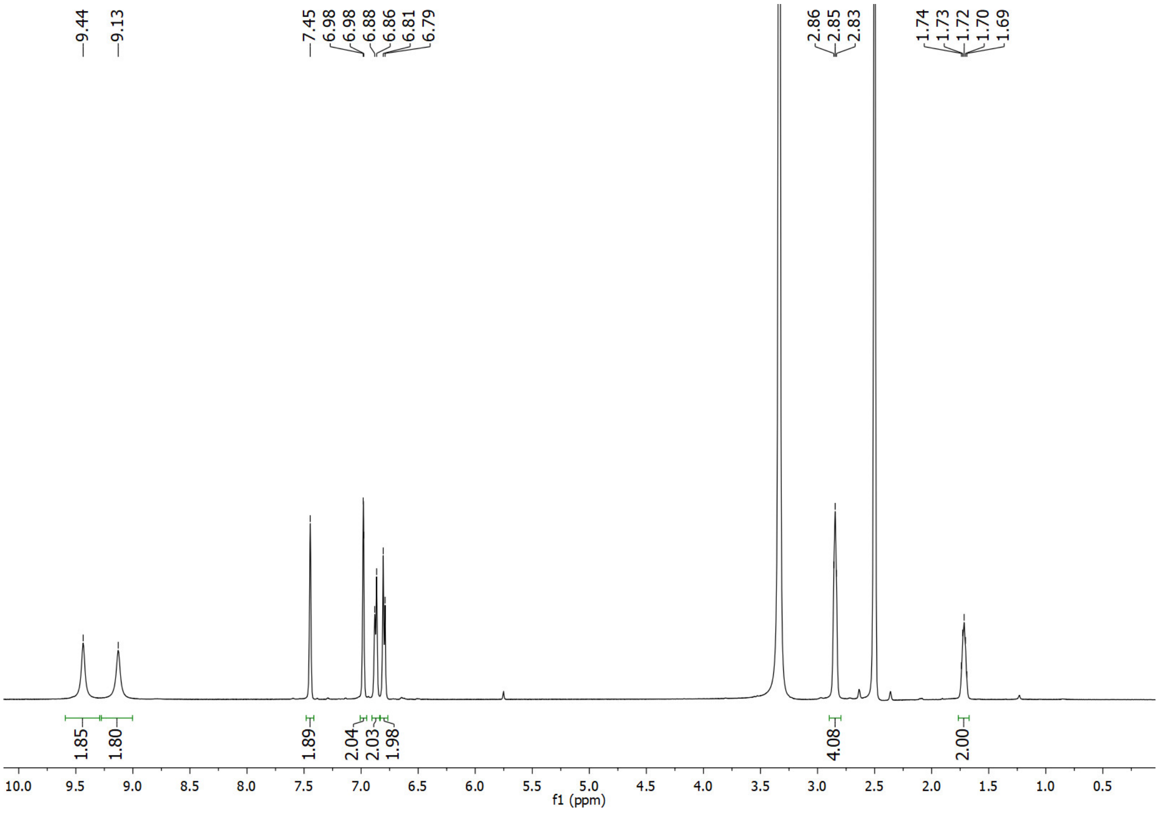

Figure S9. **^{1}H NMR spectrum** (500 MHz, DMSO-d_6, δ ppm)

(1E,4E)-1,5-bis(3,4,5-trihydroxyphenyl)penta-1,4-dien-3-one **10**

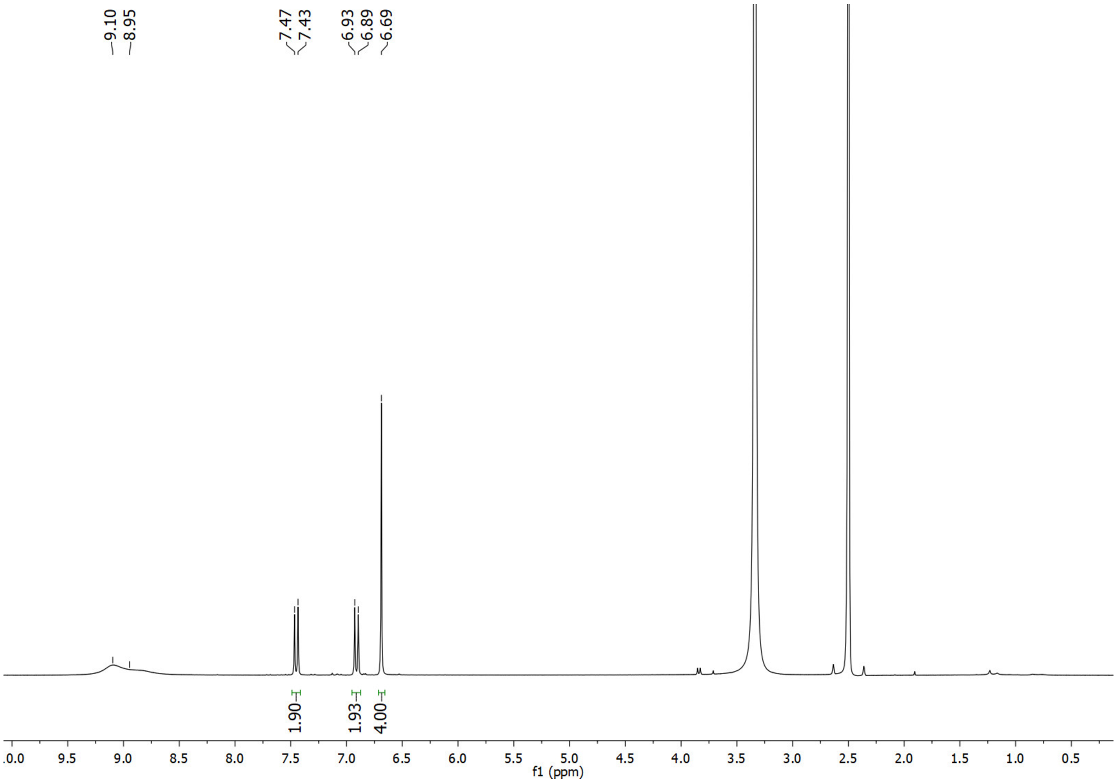

Figure S10. **^{1}H NMR spectrum** (500 MHz, DMSO-d_6, δ ppm)

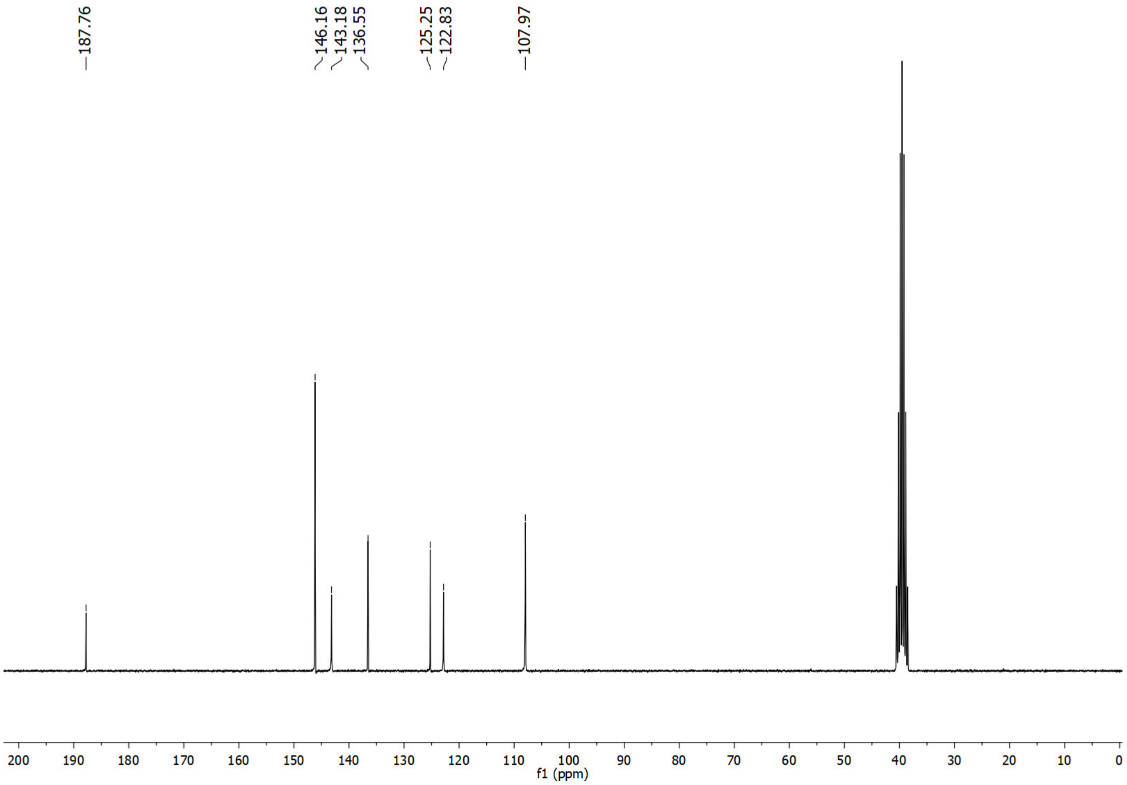

Figure S11. ^{13}C NMR spectrum (62.5 MHz, DMSO-d_6, δ ppm)

(E)-4-(3,4-dimethoxyphenyl)but-3-en-2-one **11**

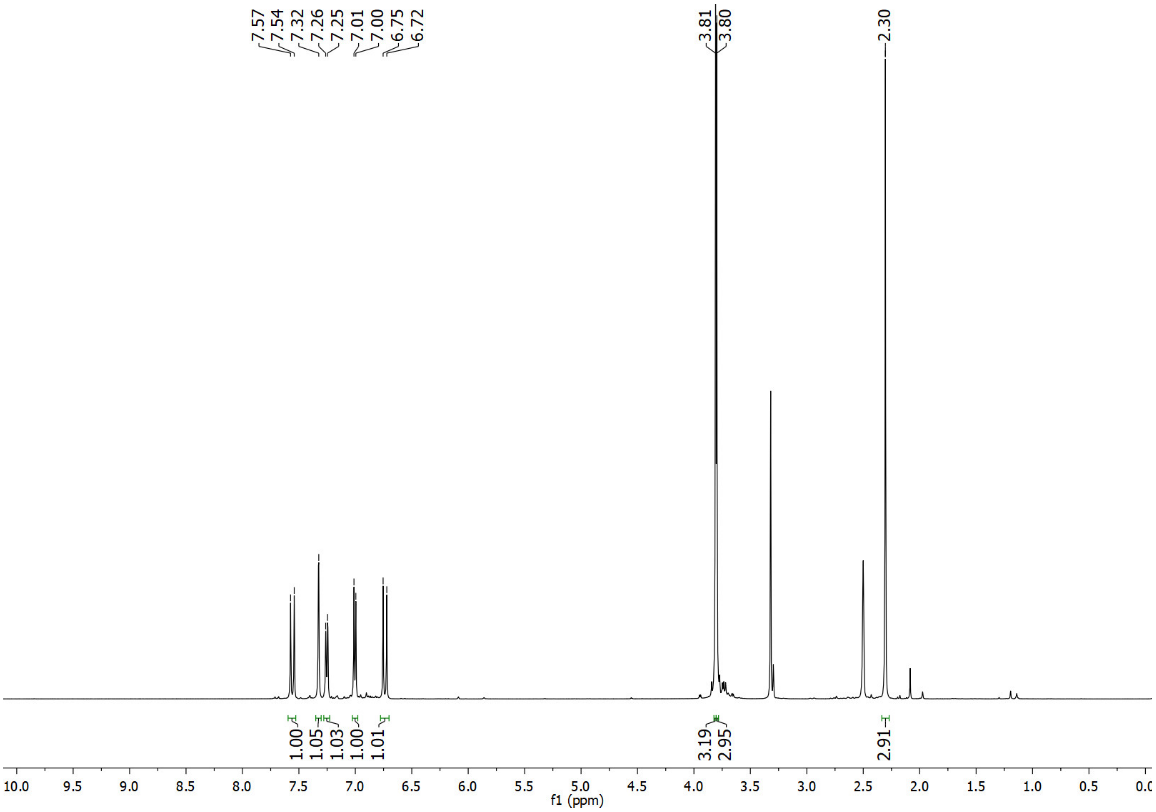

Figure S12. ¹H NMR spectrum (500 MHz, DMSO-d_6, δ ppm)

(E)-4-(4-hydroxy-3-methoxyphenyl)but-3-en-2-one **15**

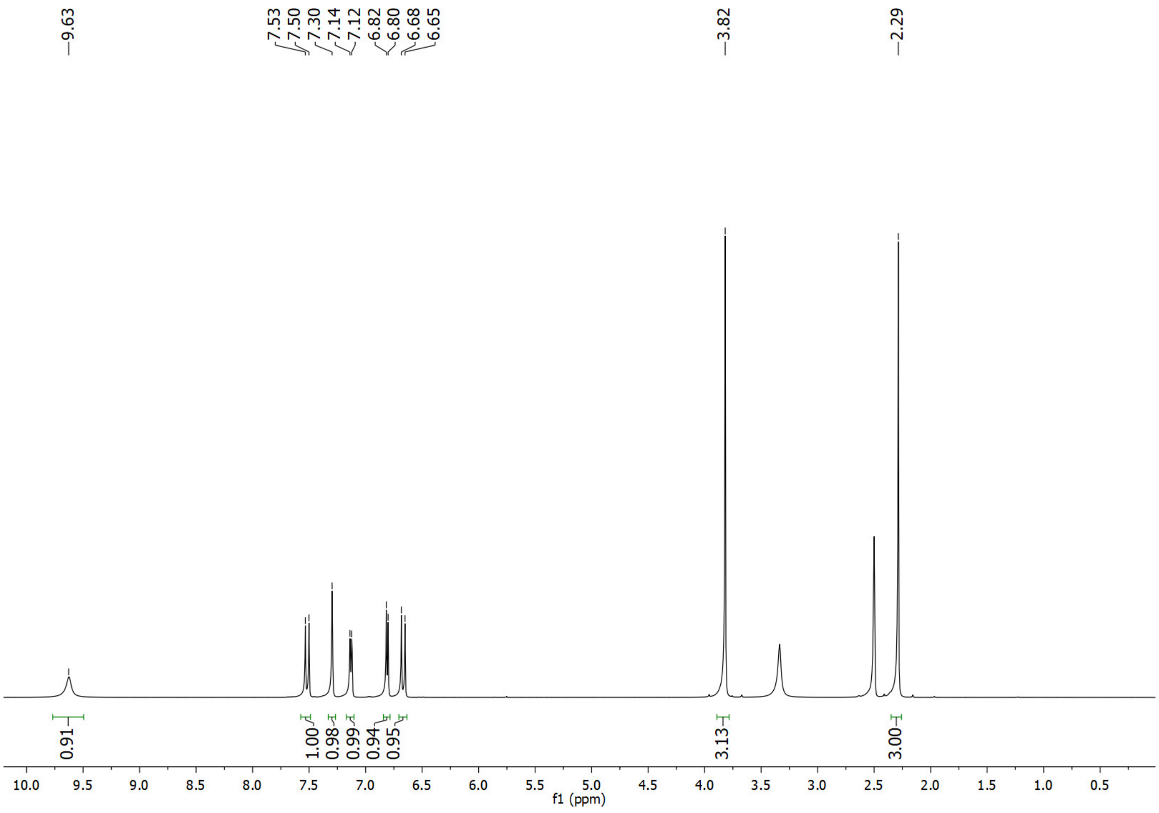

Figure S13. ^{1}H NMR spectrum (500 MHz, DMSO-d_6, δ ppm)

(E)-4-(3,4,5-trimethoxyphenyl)but-3-en-2-one **18**

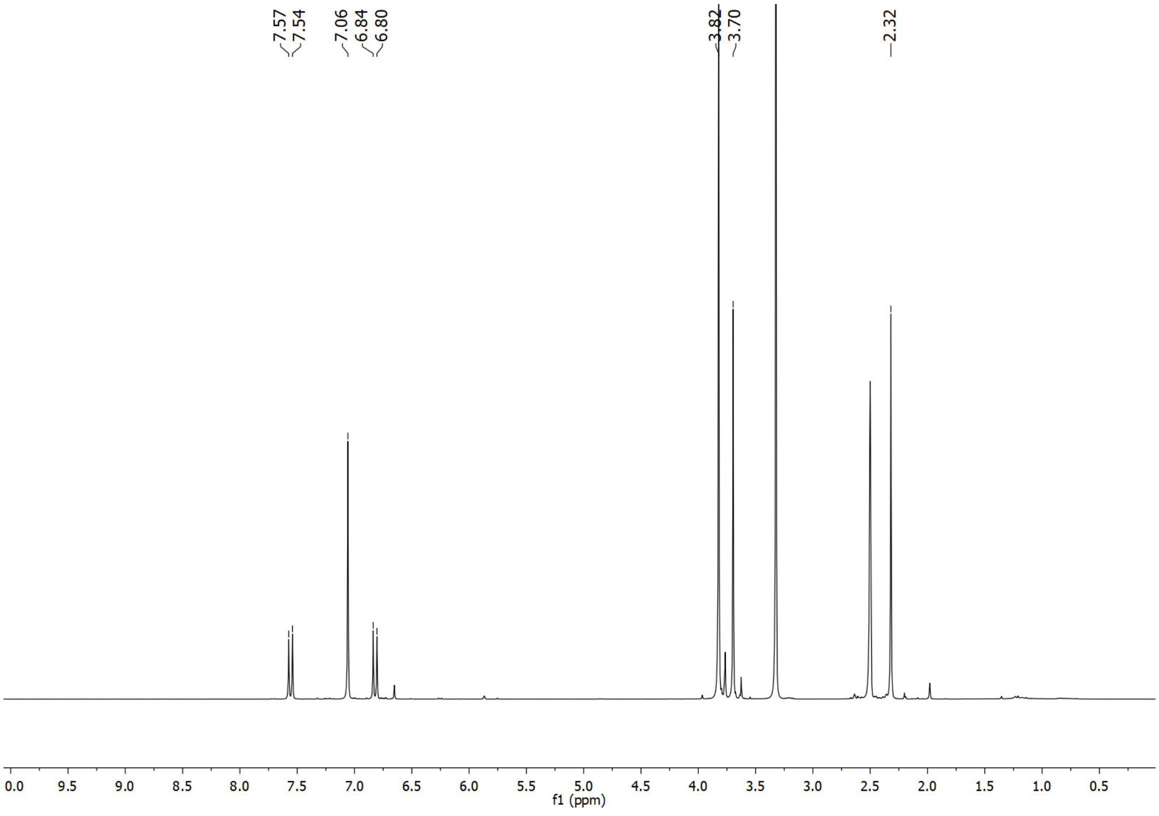

Figure S14. ¹H NMR spectrum (500 MHz, DMSO-d_6, δ ppm)

(1E,4E)-1-(3,4-dimethoxyphenyl)-5-(4-methoxyphenyl)penta-1,4-dien-3-one **12**

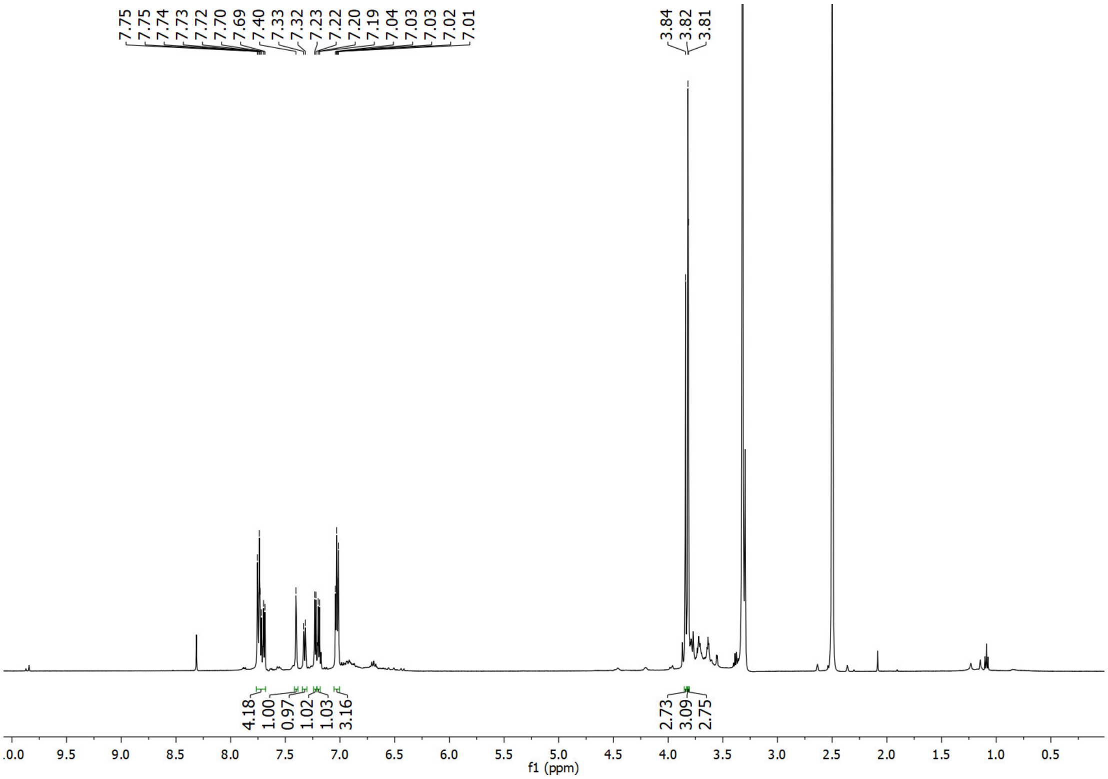

Figure S15. ¹H NMR spectrum (500 MHz, DMSO-d_6, δ ppm)

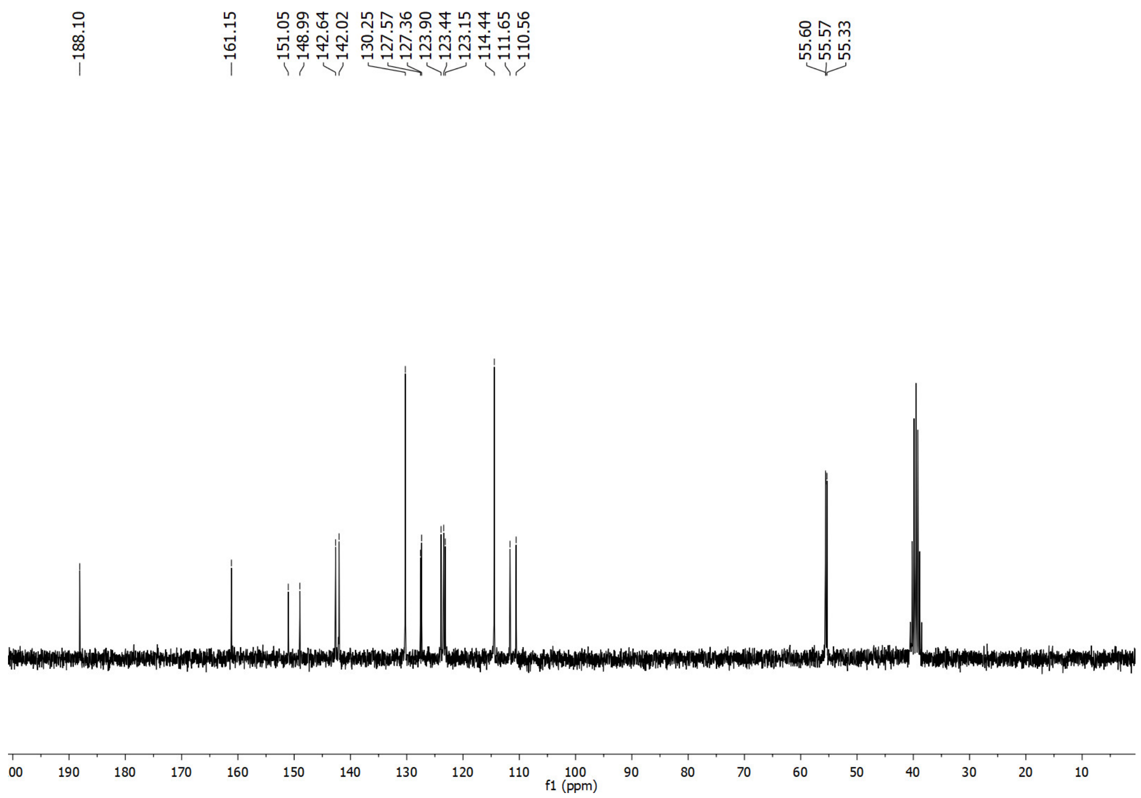

Figure S16. ^{13}C NMR spectrum (125 MHz, DMSO-d_6, δ ppm)

(1E,4E)-1-(3,4-dihydroxyphenyl)-5-(4-hydroxyphenyl)penta-1,4-dien-3-one **13**

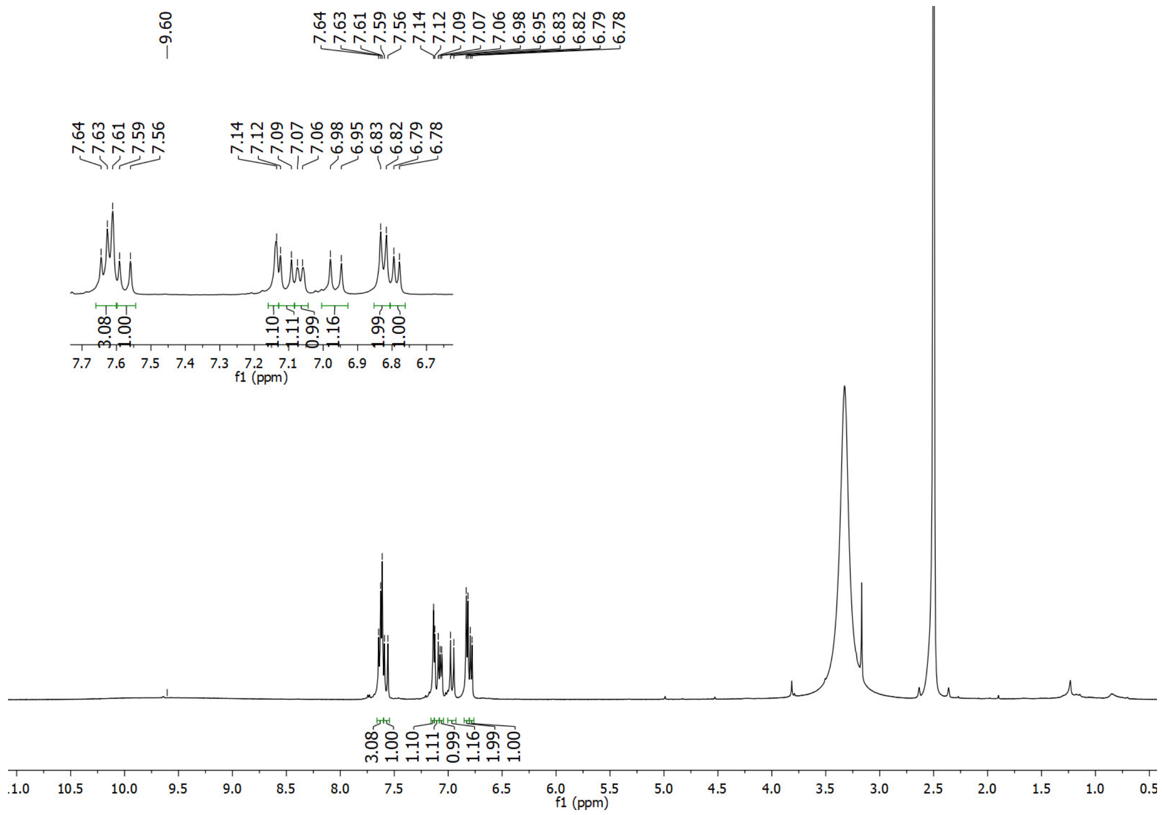

Figure S17. ^{1}H NMR spectrum (500 MHz, DMSO-d_6, δ ppm)

3,4-bis(methoxymethoxy)benzaldehyde **14**

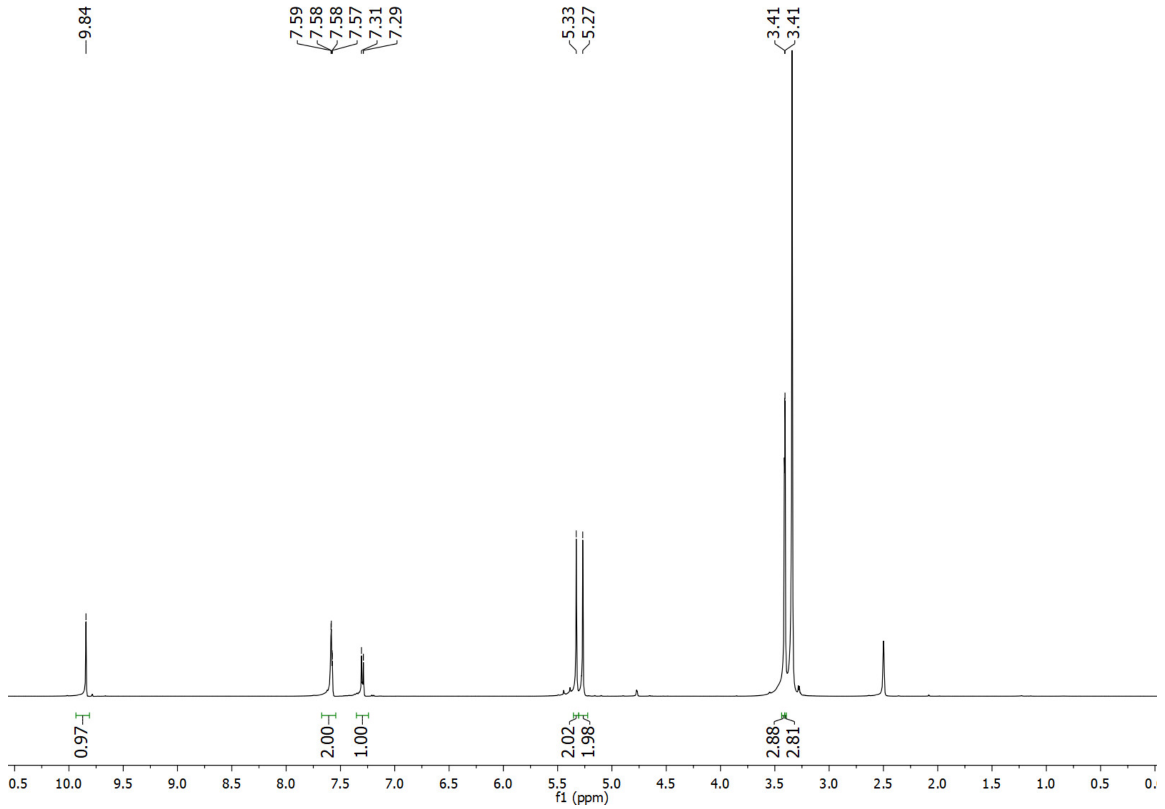

Figure S18. ^{1}H NMR spectrum (500 MHz, DMSO-d_6, δ ppm)

(1E,4E)-1-(3,4-dihydroxyphenyl)-5-(4-hydroxy-3-methoxyphenyl)penta-1,4-dien-3-one **17**

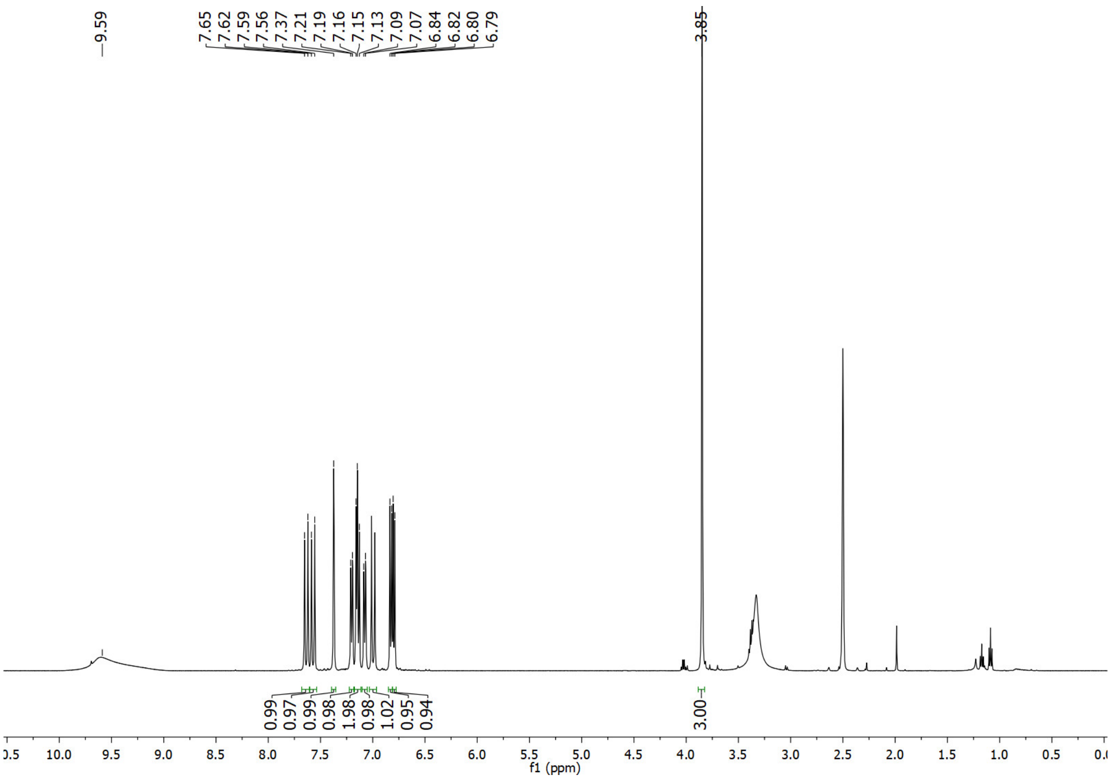

Figure S19. ^{1}H NMR spectrum (500 MHz, DMSO-d_6, δ ppm)

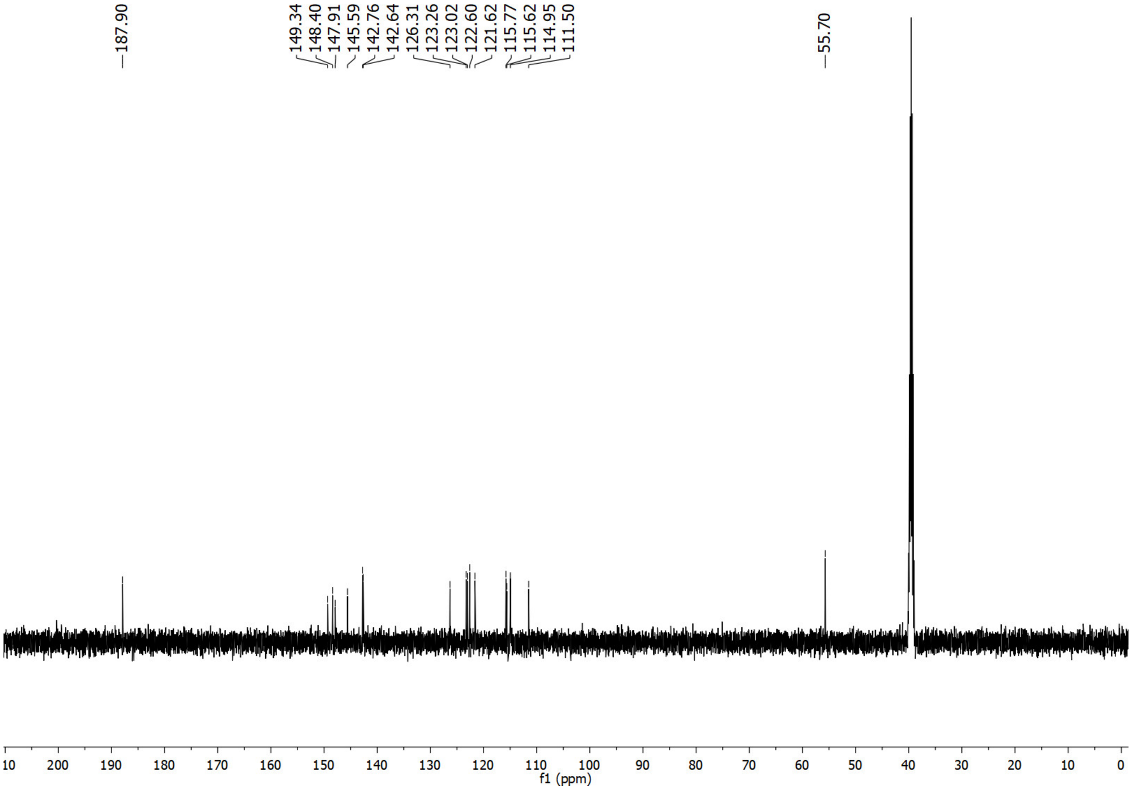

Figure S20. ^{13}C NMR spectrum (125 MHz, DMSO-d_6, δ ppm)

(E)-4-(3,4,5-trihydroxyphenyl)but-3-en-2-one **19**

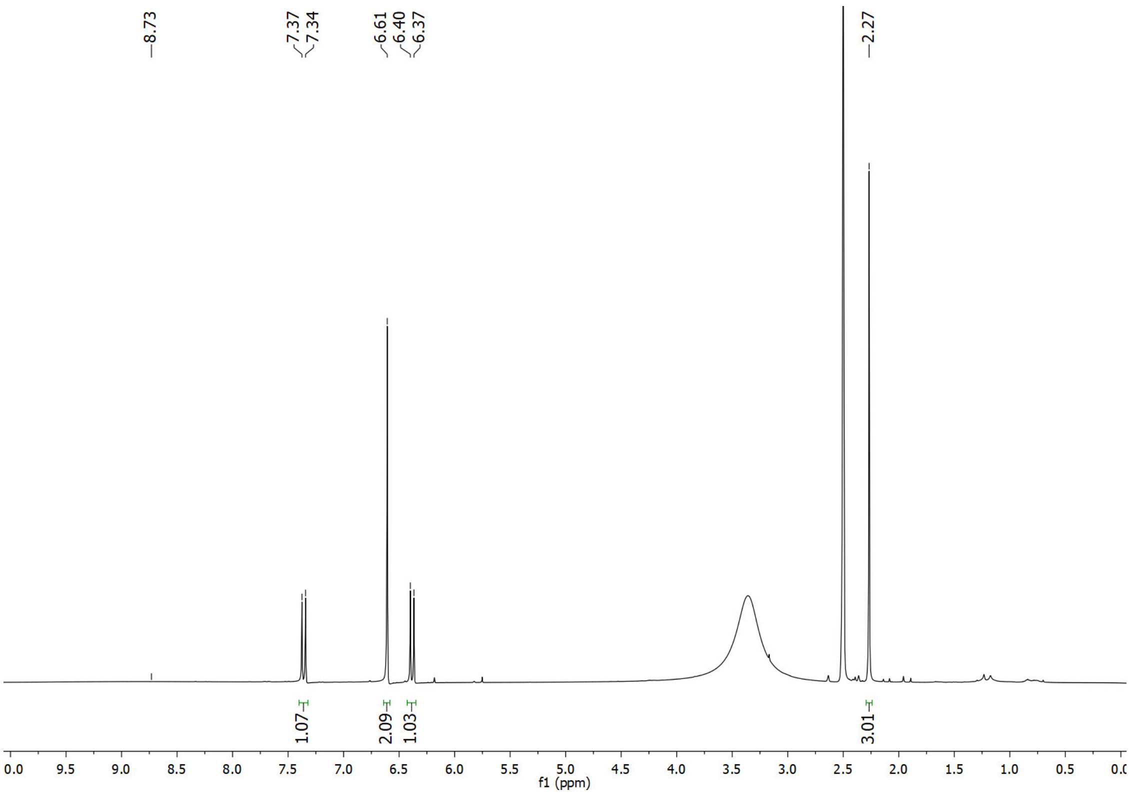

Figure S21. **^{1}H NMR spectrum** (500 MHz, DMSO-d_6, δ ppm)

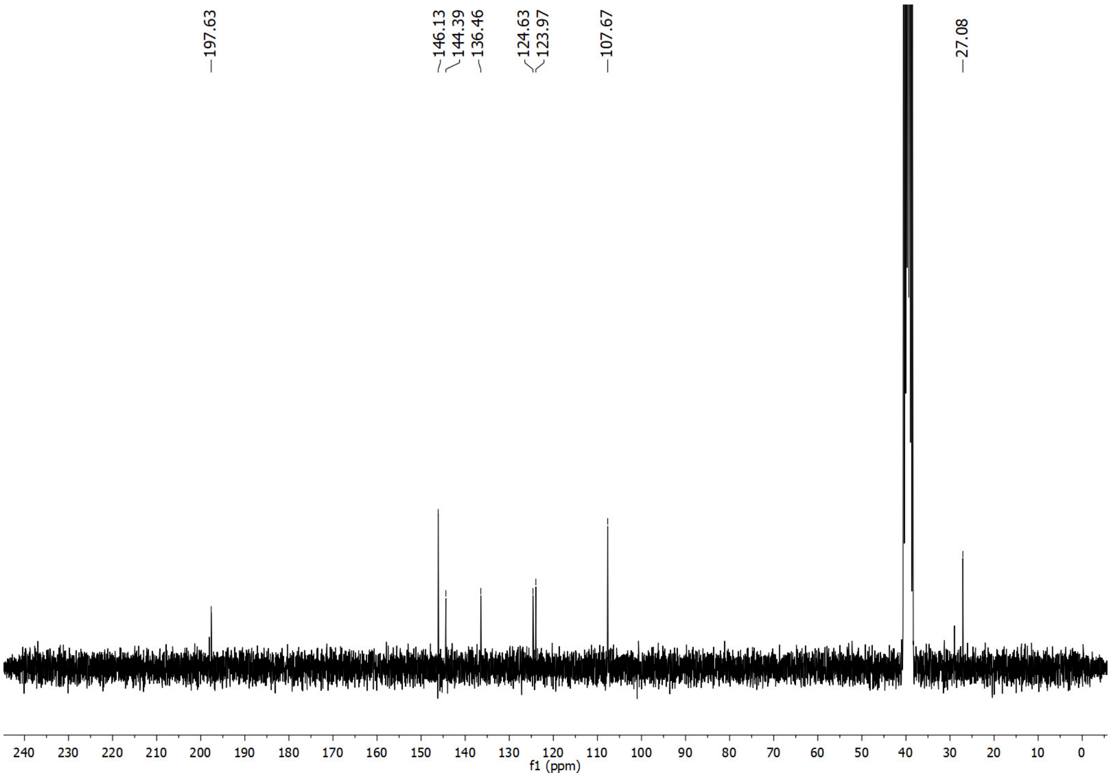

Figure S22. **^{13}C NMR spectrum** (125 MHz, DMSO-d_6, δ ppm)
